# Dynamic Coupling of Pattern Formation and Morphogenesis in the Developing Vertebrate Retina

**DOI:** 10.1371/journal.pbio.1000214

**Published:** 2009-10-13

**Authors:** Alexander Picker, Florencia Cavodeassi, Anja Machate, Sabine Bernauer, Stefan Hans, Gembu Abe, Koichi Kawakami, Stephen W. Wilson, Michael Brand

**Affiliations:** 1 Center of Regenerative Therapies Dresden (CRTD), Biotechnology Center, Dresden University of Technology, Dresden, Germany; 2 Department of Cell and Developmental Biology, University College London, London, United Kingdom; 3 Max Planck Institute of Molecular Cell Biology and Genetics, Dresden, Germany; 4 Division of Molecular and Developmental Biology, National Institute of Genetics, Mishima, Japan; 5 Department of Genetics, National Institute of Genetics, The Graduate University for Advanced Studies (SOKENDAI), Mishima, Japan; Cambridge University, United Kingdom

## Abstract

During embryonic development, pattern formation must be tightly synchronized with tissue morphogenesis to coordinate the establishment of the spatial identities of cells with their movements. In the vertebrate retina, patterning along the dorsal-ventral and nasal-temporal (anterior-posterior) axes is required for correct spatial representation in the retinotectal map. However, it is unknown how specification of axial cell positions in the retina occurs during the complex process of early eye morphogenesis. Studying zebrafish embryos, we show that morphogenetic tissue rearrangements during eye evagination result in progenitor cells in the nasal half of the retina primordium being brought into proximity to the sources of three fibroblast growth factors, Fgf8/3/24, outside the eye. Triple-mutant analysis shows that this combined Fgf signal fully controls nasal retina identity by regulating the nasal transcription factor Foxg1. Surprisingly, nasal-temporal axis specification occurs very early along the dorsal-ventral axis of the evaginating eye. By in vivo imaging GFP-tagged retinal progenitor cells, we find that subsequent eye morphogenesis requires gradual tissue compaction in the nasal half and directed cell movements into the temporal half of the retina. Balancing these processes drives the progressive alignment of the nasal-temporal retina axis with the anterior-posterior body axis and is controlled by a feed-forward effect of Fgf signaling on Foxg1-mediated cell cohesion. Thus, the mechanistic coupling and dynamic synchronization of tissue patterning with morphogenetic cell behavior through Fgf signaling leads to the graded allocation of cell positional identity in the eye, underlying retinotectal map formation.

## Abstract 2

In this Research Article, Picker et al. show how cells in the retina get their spatial coordinates.

## Author Summary

The vertebrate brain contains a point-to-point representation of sensory input from the eye. This visual map forms during embryonic development, by neuronal cells of the retina sending targeted axon projections to the brain. Since the projection needs to wire up neighboring cell positions in the retina to neighboring target areas in the brain, all retinal cells must harbor a defined spatial coordinate as prerequisite for map formation. How such a retinal coordinate system is established and maintained in the dynamically evolving embryo is a fundamental, but unresolved, question. By combining genetic analysis and in vivo imaging in zebrafish embryos, we have tracked the developmental origin of cell coordinates in the retina. We find that three related Fgf signals emanating from outside the eye define relative cell positions in the retina very early, already at the onset of its formation. But the absolute position of retinal cells relative to the body axes is greatly rearranged during subsequent development. In this phase, surprisingly, the same Fgf signals that at first defined retinal cell positions now balance asymmetric cell movements and cell shape changes, which are required for harmonic retinal growth and the final alignment of cell coordinates in the eye.

## Introduction

Map-like representation of sensory information is an evolutionary conserved principle of brain organization and function [1]. The point-to-point mapping of retinal ganglion cell (RGC) axons onto the midbrain tectum/superior colliculus of the vertebrate, is a hallmark example for the requirement of precise pattern formation during embryonic development, since mapping occurs according to the position of RGCs along the nasal-temporal (anterior-posterior) and dorsal-ventral axes of the retina. The topographic projections of RGC axons accurately preserve information on cell positions and neighborhood relationships in the retina as a continuous map of terminals in the tectum [2].

Cell-surface axon guidance molecules expressed in gradients across the retina and tectum control the formation of retinotopic connections [3]–[8]. Guidance molecule expression along the nasal-temporal retina axis is regulated by the nasal- and temporal-specific transcription factors Foxg1, Foxd1, SOHo, and GH6 [9]–[12]. However, expression of these factors in the retina is asymmetrical from the onset, indicating that they act downstream of nasal-temporal axis specification. Retinotopic mapping consequently occurs as a function of RGC position along molecular gradients within a coordinate system set by the major retinal axes. This suggests that axis formation and mapping are intimately connected developmental processes, but the nature and timing of the signals that establish cell positional identities in this coordinate system are largely unknown.

Resolving the mechanisms underlying the allocation of positional identity to retinal cells is confounded by the complex morphogenetic rearrangements of forebrain tissues that occur during eye formation [13]–[16]. Morphogenesis of the retina begins with the lateral displacement of cells in the eye field to the site of future optic vesicle evagination [17],[18]. Subsequently, cells continuously evaginate from the forebrain, steadily increasing the size of the optic vesicle. Next, the optic vesicle invaginates to form the two-layered optic cup, with the outer layer, which faces the surface ectoderm and lens, fated to become neural retina and the inner layer the retinal pigment epithelium. Cell movements from the presumptive pigmented epithelium into the neural retina may occur during this phase [19]. Later optic cup development depends on integrin-mediated focal adhesion at the basal side of the retinal epithelium [20]. However, although the anatomy of the optic cup is well described, dynamic in vivo analysis of its formation is lacking, and axial patterning of the prospective retina has not been studied prior to the completion of optic cup formation [21],[22]. It thus remains to be determined how and when axial cell positions in the retina are specified and how the orientation of the retinal axes is related to the axes of the neural tube during eye morphogenesis.

Fgfs have conserved functions in axial patterning of the neural tube. Originating from local organizers, they can control cell positional identities in adjacent regions, with several Fgfs often acting in a combinatorial manner [23],[24]. We have previously shown that Fgf8 signaling contributes to nasal-temporal patterning of the retina, and proposed a combinatorial Fgf signal to exert full control over the specification of this retinal axis [25], but the nature of the Fgfs involved, their dynamic requirement, and sites of action during eye morphogenesis are not resolved.

We show here that a combined Fgf8/3/24 signal specifies the positional identities of cells along the nasal-temporal retinal axis in zebrafish embryos. Absence of all three factors leads to completely temporalized, and ectopic activation of Fgf signaling to completely nasalized retinae. Axis specification occurs very early, at the onset of optic vesicle evagination, when *fgf8/3* are expressed in the dorsal forebrain and *fgf24* in the dorsally located olfactory placode. During formation of the optic vesicle, Fgf signaling from these dorsal sources is required to confine expression of *foxg1* (and other future nasal genes) to the dorsal half, and *foxd1* (and other temporal genes) to the ventral half of the evaginating optic vesicle. Thus, at the moment of specification, nasal-temporal cell positional identities align parallel to the dorsal-ventral axis of the neural tube and asymmetrically relative to the dorsal sources of Fgf.

By in vivo tracking of GFP-labeled nasal and temporal retina progenitor cells in transgenic lines, we further show how tightly synchronized morphogenetic cell movements and cell shape changes during optic cup formation lead to axis reorientation and the final nasal-temporal subdivision of the neural retina. This occurs as the result of two temporally concordant morphogenetic processes: (1) compaction of nasal retina progenitors already residing in the future neural retina domain—possibly by shortening along the lateral and elongation along the apical-basal cell axis—and (2) directed movement of temporal retina progenitors into that domain. In this process, Foxg1 promotes cohesion of nasal progenitor cells in an Fgf-dependent manner, thereby probably allowing the gradual addition of temporal progenitors to the growing neural retina. Thus, the dynamic coordination of pattern formation with neuroepithelial morphogenesis through Fgf8/3/24 signaling controls the final arrangement of axial cell positions in the retina.

## Results

### Combined Fgf8/3/24 Signaling Controls Nasal-Temporal Patterning of the Retina

Fgf8 is involved in nasal-temporal patterning of the retina, but loss of *fgf8* results only in subtle patterning defects, demonstrating the presence of other unknown factors controlling this process [25]. To test whether Fgf8 acts in combination with other Fgfs, we studied the expression of nasal and temporal marker genes in embryos that lack two or more Fgfs.

**Figure 1:**
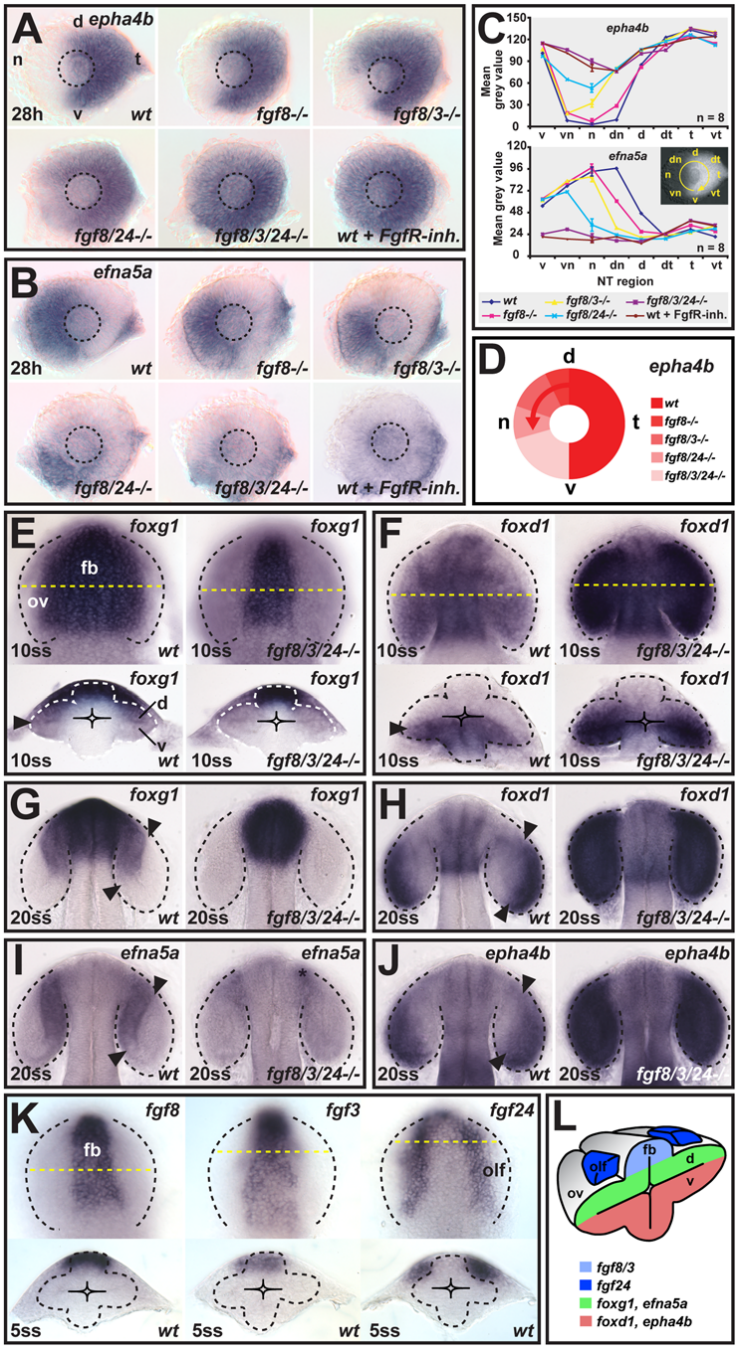
*fgf8/3/24* impose nasal-temporal pattern by signaling along the dorsal-ventral axis of the optic vesicle. (A and B) Expression of (A) *epha4b* in the temporal and (B) *efna5a* in the nasal retina of wild-type (wt) control, *fgf8* mutant (*fgf8^−/−^*), *fgf8/3* double mutant (*fgf8/3^−/−^*), *fgf8/24* double mutant (*fgf8/24^−/−^*), *fgf24*-morpholino injected *fgf8/3* double mutant (*fgf8/3/24^−/−^*), and wt embryos after Fgf receptor inhibitor treatment (wt+FgfR-inh.) at 28 h (nasal is to the left and dorsal is up). Dashed-line circle indicates position of the lens. (C) Nasal-temporal profile of *epha4b* and *efna5a* gene expression levels (mean grey value for *n* = 8 images analyzed for each genotype, *y*-axis, in defined nasal-temporal (NT) regions of the retina, *x*-axis, see inset, one representative standard deviation is plotted). (D) Schematic illustrating the graded, nasal expansion of the temporal marker *epha4b* in the phenotypic series of *fgf* mutants. (E and F) Expression of the future nasal marker *foxg1* (E) and the future temporal marker *foxd1* (F) at 10ss in the optic vesicle (ov) of wt (left) and *fgf8/3/24^−/−^* embryos (right) (top: dorsal view, anterior to the top, bottom: cross-section, dorsal to the top). (G–J) Expression of the future nasal markers *foxg1* (G) and *efna5a* (I) and the future temporal markers *foxd1* (H) and *epha4b* (J) in the optic cup of wt (left) and *fgf8/3/24^−/−^* (right) embryos at 20ss (dorsal views, anterior to the top). Asterisk in (I): remnant *efna5a* expression in the optic stalk region. (K) Expression of *fgf8* and *fgf3* in the dorsal forebrain (fb), and *fgf24* in the olfactory placode (olf) in wt embryos at 5ss. Orientation as in (E). (L) Schematic cross-section, illustrating dorsal expression of *fgf*s and expression of future nasal-temporal retina markers along the dorsal-ventral optic vesicle axis at 10ss. Arrowheads in (E–J): gene expression limits in the optic vesicle and cup. Yellow dotted lines indicate transverse section level. Black and white dotted lines indicate neural tube or optic vesicle/cup outlines. d, dorsal; dn, dorsonasal; dt, dorsotemporal; n, nasal; t, temporal; v, ventral; vn, ventronasal; vt, ventrotemporal.

We find that *fgf3* and *fgf24* strongly interact with *fgf8* in nasal-temporal patterning (Figure 1A and 1B). In wild-type (wt) control embryos at 28 h, the Eph receptor *epha4b* is expressed in a temporal-to-nasal decreasing gradient, and the ephrin ligand *efna5a* in a complementary nasal-to-temporal decreasing gradient. In *fgf8^−/−^* mutants, *epha4b* expression expands into the dorsonasal retina, and *efna5a* expression is reduced in that region. This phenotype is enhanced in *fgf8/3^−/−^* double mutants; and in *fgf8/24^−/−^* double mutants, the changes in *epha4b* and *efna5a* expression are even stronger, now also affecting the ventronasal retina. Upon inactivation of all three *fgf*s, by *fgf24*-morpholino injection in *fgf8/3^−/−^* double mutants (referred to as *fgf8/3/24^−/−^*), all retinal cells express *epha4b* and none *efna5a*. The same result is obtained upon blocking of all Fgf receptor signaling with a pharmacological inhibitor (FgfR-inh.) between the 1- and 5-somite stages (ss). *Fgf3^−/−^* and *fgf24^−/−^* single-mutants have no detectable retinal patterning or eye defects (unpublished data).

Analysis of *efna5a* and *epha4b* expression levels in defined axial regions of the retina shows that stepwise elimination of *fgf8/3* and *-24* results in a graded phenotypic series: wt control (normal nasal-temporal pattern)<*fgf8^−/−^*<*fgf8/3^−/−^*<*fgf8/24^−/−^*<*fgf8/3/24^−/−^* or FgfR-inh.–treated embryo (*all temporal* pattern) (Figure 1C). The requirement for *fgf8/3/24* is more pronounced in the dorsonasal than in the ventronasal retina, as illustrated by the stepwise temporal-to-nasal expansion of *epha4b* (Figure 1D). Thus, Fgf8/3/24 constitute a combined Fgf signal that fully controls nasal-temporal patterning of the neural retina.

### Nasal-Temporal Pattern Is First Evident along the Dorsal-Ventral Axis of the Optic Vesicle

The eye undergoes highly complex morphogenetic movements during its evagination, and the locations of cells contributing to the future nasal and temporal retina have not been followed during this process. To investigate the initial orientation of the retinal axes, we studied the early expression of genes that are later restricted along the nasal-temporal retina axis. In wt 10ss embryos, the future nasal marker, *foxg1*, is expressed in the dorsal leaflet of the evaginating optic vesicle (Figure 1E, left), whereas the future temporal marker, *foxd1*, (see Materials and Methods and Figure S11 and S12 for *foxd1* gene nomenclature) is expressed in the ventral optic vesicle leaflet (Figure 1F, left). Surprisingly, nasal-temporal markers, therefore, initially align with the dorsal-ventral axis of the neural tube. By 20ss, the future nasal markers, *foxg1* and *efna5a*, are restricted to the anterior-medial optic cup (Figure 1G and 1I, left), whereas the future temporal markers *foxd1* and *epha4b* are expressed in a complementary posterior-lateral domain (Figure 1H and 1J, left). From 24 h, these markers are expressed in the anterior nasal half and posterior temporal half of the retina (Figure 1A and 1B and unpublished data). Time-lapse imaging of transgene-labeled retinal cells during optic cup morphogenesis supports the conclusion from these gene expression analyses that the dorsal-ventral axis of the optic vesicle corresponds to the later nasal-temporal axis of the retina (see below).

### Fgf Activity Imposes Nasal-Temporal Pattern during Optic Vesicle Evagination

To determine when Fgfs impose nasal-temporal identity, we analyzed early nasal-temporal markers in embryos lacking Fgf8/3/24. In 10ss *fgf8/3/24^−/−^* embryos, *foxg1* expression is absent from the dorsal optic vesicle leaflet (Figure 1E, right) and *foxd1* is expanded throughout the vesicle (Figure 1F, right), indicating a complete nasal-to-temporal patterning shift. Similarly, in 20ss *fgf8/3/24^−/−^* embryos, no *foxg1*/*efna5a* expression is detectable in the optic cup. (Figure 1G and 1I, right) and *foxd1*/*epha4b* expression is expanded throughout the optic cup (Figure 1H and 1J, right). This shows that Fgf8/3/24 control nasal-temporal patterning of the retina at the onset of optic vesicle evagination.

We next addressed the source of the Fgfs that pattern the optic vesicle along its dorsal-ventral axis, the future nasal-temporal axis of the retina. Early, spatially restricted *fgf8/3/24* expression relative to the domains of nasally/temporally expressed genes explains the patterning activity of Fgfs along the dorsal-ventral axis of the optic vesicle (Figure 1K). At 5ss, towards the end of the requirement phase for Fgf signaling in nasal-temporal patterning, *fgf8* and -*3* are expressed in the dorsal forebrain, which is contiguous with, and close to, the *foxg1*-expressing, dorsal optic vesicle leaflet and distant to the *foxd1*-expressing, ventral leaflet. *fgf24* is expressed in cells of the nascent olfactory placode [26], at the hinge between the dorsal forebrain and the dorsal optic vesicle leaflet. *Fgf24*-expressing cells remain in close contact with the developing optic vesicle and cup during later morphogenesis (Figure S1). Thus, expression of future nasal markers occurs close to the dorsal source of Fgfs and expression of future temporal markers distant to it (Figure 1L). Local and graded Fgf signaling in the dorsal optic vesicle leaflet is further supported by nested, Fgf-dependent expression of the Fgf pathway target genes *erm*, *pea3*, *spry2*, and *spry4* (Figure S2).

### Fgf Signaling Enhances Proliferation of Nasal Retina Progenitors

Since Fgfs are known mitogens and Foxg1 promotes neural progenitor proliferation [27]–[30], we assessed whether the nasal-temporal asymmetry in Fgf signaling affects cell proliferation in the optic vesicle.

**Figure 2:**
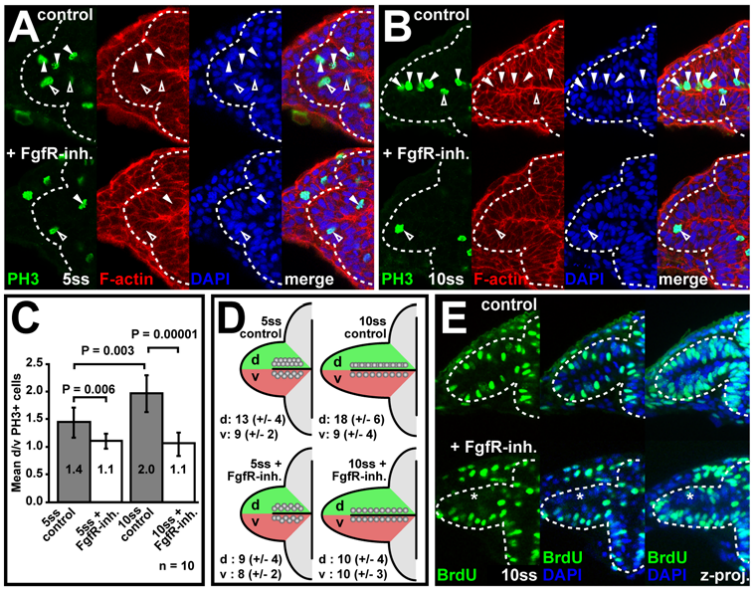
Fgf signaling controls enhanced nasal retina progenitor proliferation. (A and B) Detection of mitotic cells with anti-Phospho Histone H3 (PH3, green), counterstained for cortical F-actin (red) and cell nuclei with DAPI (blue) in the optic vesicle of carrier-treated wt control (top) and FgfR-inh.-treated embryos (bottom) at 5ss (A) and 10ss (B) (filled arrowheads: dorsal leaflet cells, open arrowheads: ventral leaflet cells). (C) Quantification of PH3^+^ cell numbers in the optic vesicle. Mean ratios of mitotic cells in the dorsal (d) and ventral (v) optic vesicle leaflets (mean d/v PH3+ cells, *y*-axis) for control (grey) and FgfR-inh.-treated embryos (white) at 5- and 10ss (error bars: standard deviation). (D) Schematic plotting of the mean number of PH3^+^ cells/optic vesicle dorsal and ventral leaflet (complete anterior-posterior extent of the optic vesicle) into one plane (±standard deviation in brackets). (E) Anti-BrdU staining (green) to detect the proliferation pattern in the optic vesicle at 10 ss in carrier-treated wt control (top) and FgfR-inh.-treated embryos (bottom). Cell nuclei are counterstained with DAPI (blue) (asterisks: reduction in BrdU incorporation, left and middle: single optical sections, right: *z*-projection). Orientation: cross-sections through one half of the forebrain, dorsal to the top and lateral to the left. Dotted lines in (A, B and E): neural tube boundary.

Control embryos, stained for the mitosis marker Phospho-Histone H3 (PH3), show slightly more PH3-positive cells in the dorsal than the ventral optic vesicle leaflet at 5ss (Figure 2A, top). This asymmetry becomes clearer at 10ss (Figure 2B, top), when the apical side of the dorsal leaflet is often densely populated by PH3-positive cells, a pattern never observed in the ventral leaflet. In FgfR-inh.–treated embryos, this asymmetric proliferation pattern is lost (Figure 2A and 2B, bottom). Counting and plotting of the mean ratios of dorsal/ventral leaflet PH3-positive cells shows the increasing asymmetry in proliferation in control embryos and its loss upon FgfR-inhibition: at 10ss, 2-fold more dividing cells are found in the dorsal than in the ventral leaflet in control embryos, whereas the ratio is near 1∶1 after FgfR inhibition (Figure 2C). Analysis of the mean PH3-positive cell number per optic vesicle leaflet, shows that progenitor proliferation is selectively affected in the nasal retina primordium/dorsal optic vesicle leaflet after FgfR inhibition, whereas proliferation of temporal retina progenitors in the ventral optic vesicle leaflet is unchanged (Figure 2D). Similarly, BrdU incorporation at 10ss is severely reduced in nasal progenitors of the dorsal optic vesicle leaflet upon FgfR inhibition. Treatment has no obvious effect on temporal progenitors in the ventral leaflet (Figure 2E). Thus, Fgf signaling is selectively required for enhanced proliferation of nasal retinal progenitors during optic vesicle evagination, and this requirement coincides with the Fgf-dependent regulation of *foxg1* in the dorsal optic vesicle leaflet.

### Directed Movements of Temporal Retina Progenitor Cells into the Optic Cup

**Figure 3:**
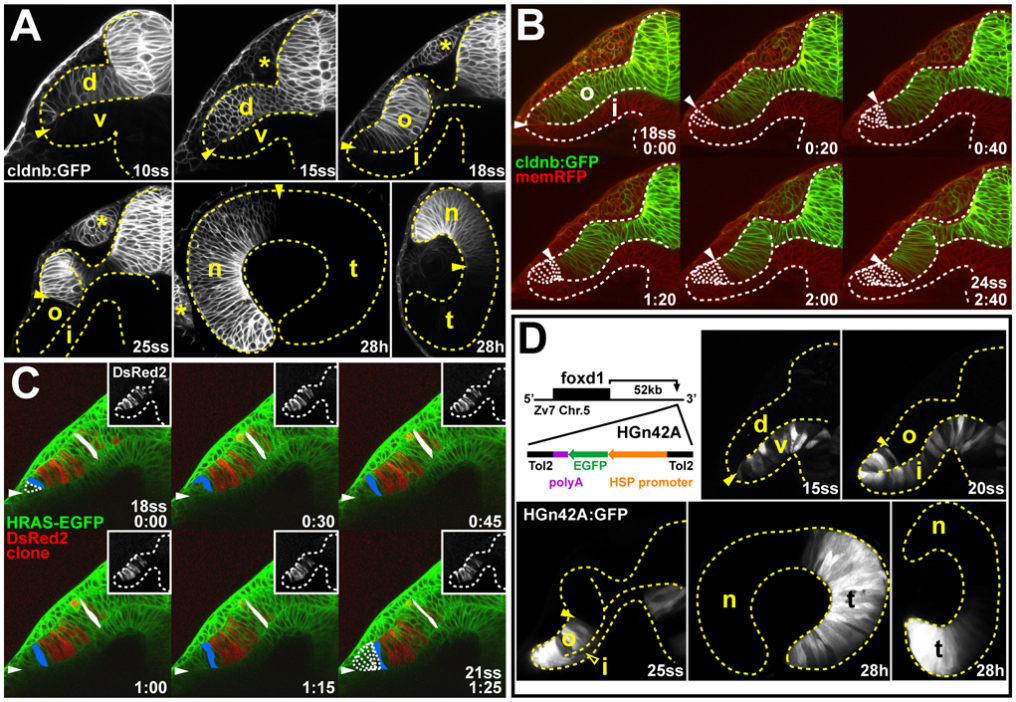
In vivo imaging reveals late movement of prospective temporal retinal cells around the distal ridge of the optic cup. (A) GFP expression (white) in the optic vesicle and optic cup of live *Tg(-8.0cldnb:lynGFP)zf106* zebrafish embryos at 10ss, 15ss, 18ss, and 25ss (cross-sections through one half of the forebrain, lateral is to the left) and 28 h (longitudinal section with nasal to the left and horizontal section with nasal to the top). Arrowheads: cldnb:GFP expression limit. (B) Single images from time-lapse analysis of *Tg(-8.0cldnb:lynGFP)zf106* expression (green) between 18- (late optic vesicle) and 24ss (early optic cup), colabeled with membrane RFP (memRFP, red), at 20-min intervals. cldnb:GFP^−^ cells moving from the inner optic cup layer, around the distal optic cup ridge are outlined (arrowheads: distal cldnb:GFP expression limit, bottom right: time in hours∶minutes). (C) Single images from a time-lapse analysis to track a DsRed2-expressing cell clone (red, insets: single channel) in the outer optic cup layer of a *Tg(Bactin:HRAS-EGFP)vu119* host embryo, expressing membrane-targeted GFP (green) between 18- and 21ss, at 30-min intervals. Cells moving from the inner optic cup layer around the distal optic cup ridge are outlined in the first and last image of the series. One cell at the distal limit of the clone (blue) and one cell at the dorsal limit of the clone (white) are pseudocolored (maximal outlines of colored cells and representative apical-basal axis measurements are based on confocal *z*-stack projections of the complete clone). Arrowheads: distal optic cup ridge, bottom right: time in hours∶minutes). (D) Transposon insertion site in *HGn42A*, 52-kbp downstream of *foxd1* on chromosome 5 (Zv7 assembly of the zebrafish genome). GFP expression (white) in the optic vesicle and optic cup of live *HGn42A* zebrafish embryos at 15ss, 20ss, and 25ss (cross-sections through one half of the forebrain, lateral is to the left) and 28 h (longitudinal section with nasal to the left and horizontal section with nasal to the top) (filled arrowheads: distal GFP expression limit, open arrowhead: GFP expression in inner optic cup layer). d, dorsal optic vesicle leaflet; i, inner optic cup layer; n, nasal; o, outer optic cup layer; t, temporal; v, ventral optic vesicle leaflet. *: olfactory placode. Orientation in (B and C): cross-sections through one half of the forebrain, dorsal to the top and lateral to the left. Dotted lines: neural tube or retina boundaries.

The unexpected initial alignment of future nasal-temporal markers along the dorsal-ventral axis raised the question how the nasal-temporal axis reaches its final anterior-posterior orientation. We thus analyzed the dynamic development of the nasal-temporal axis by in vivo imaging of eye formation in transgenic *Tg(-8.0cldnb:lynGFP)zf106* embryos [31], which we find express GFP in the nasal retina throughout development (Figure 3A and S3, see Materials and Methods). From 10- to 15ss, cldnb:GFP is expressed throughout the dorsal optic vesicle leaflet, but between 18- and 25ss, cldnb:GFP expression becomes progressively restricted to the dorsal half of the outer layer of the optic cup. The portion of the optic vesicle that contacts the lens ectoderm will form the neural retina, and henceforth, we use the term *outer layer* to describe this part of the forming optic cup. At 28 h, after completion of optic cup morphogenesis, cldnb:GFP is restricted to the nasal half of the neural retina.

To investigate the mechanism that gradually restricts cldnb:GFP expression to the dorsal half of the outer layer of the optic cup, we performed time-lapse imaging between 18- and 24ss (Figure 3B and S4). cldnb:GFP expression initially reaches the distal limit of the optic cup, which we term the *ridge*, but within about 2.5 h, its distal limit is approximately nine cell diameters from the ridge. Time-lapse movies show pronounced outward cell movement in the inner layer of the optic cup towards the distal ridge (Video S1). This suggests a gradual displacement of cldnb:GFP-positive, nasal retina progenitor cells in the outer optic cup layer—the future neural retina domain—by a late movement of cldnb:GFP-negative cells, presumably from the inner layer, around the optic cup ridge.

To determine whether outer layer cells are indeed displaced in this way, we followed the movement of DsRed2-expressing outer layer cell clones in transgenic membrane-GFP *Tg(Bactin:HRAS-EGFP)vu119* embryos [32]. A representative cell-tracking experiment shows how a cell in the ridge region (blue) of the outer optic cup (distance to ridge at 0 min: one cell diameter) is gradually displaced dorsally and proximally (distance to ridge at 1 h 25 min: six cell diameters), and as this happens, it elongates along the apical-basal axis (apical-basal axis at 0 min: 22 µm, at 1 h 25 min: 41 µm) (Figure 3C and Video S2). Notably, this lateral displacement within the optic epithelium occurs with the same kinetics as the displacement of the distal limit of cldnb:GFP expression (compare Figure 3B to 3C). A representative cell positioned further dorsally (white) is barely displaced laterally and elongates only slightly (apical-basal axis at 0 min: 34 µm, at 1 h 25 min: 45 µm) (Figure 3C). At 36 h, the clone in Figure 3C, which initially covered the complete extent of the outer optic cup layer (see insets in Figure 3C), is restricted to the nasal hemiretina (Figure S5; *n* = 5/5 analyzed outer layer clones) confirming that outer layer cells are all initially destined for nasal retina. This suggested that cell movements from the inner optic cup layer around the distal ridge region gradually add nonnasal retina progenitors to the outer layer of the optic cup. This addition of cells occurs coincident with the elongation of the apical-basal axis of nasal progenitors already residing in the outer optic cup layer, suggesting a gradual compaction of the future neural retina epithelium.

The gradual encroachment of GFP expression into the outer layer in the *HGn42A* enhancer trap line [33] is complementary to the restriction in cldnb:GFP expression (Figure 3D). The insertion in *HGn42A* maps to a site 52-kbp downstream of the *foxd1* locus, and GFP expression in this line recapitulates endogenous *foxd1* expression in the prospective temporal retina (Figure S6). These results support the conclusion that HGn42A:GFP-positive, prospective temporal, retinal cells move around the distal optic cup ridge and displace the cldnb:GFP-positive nasal progenitors to the dorsal-proximal optic cup. Thus, the nasal-temporal axis of the retina is established by Fgf-dependent patterning of the optic vesicle along the dorsal-ventral axis of the neural tube. Only later, during optic cup formation, do temporal retina progenitors start to move into the definitive neural retina domain, while nasal retina progenitors already residing there regress and compact. Concomitant with anterior eye rotation, this leads to the final alignment of the nasal-temporal retina axis with the anterior-posterior body axis.

### Nasal Retina Progenitors Delaminate from the Optic Vesicle Upon Loss of FgfR Signaling

*Fgf8/3/24^−^* ^/*−*^ embryos form smaller, but otherwise morphologically normal, retinae (Figure S7) despite the complete loss of nasal-temporal polarity and reduced proliferation, suggesting global eye morphogenesis is not compromised in the absence of nasal-temporal patterning. However, we do find that Fgf signaling specifically and regionally affects epithelial cell morphology and behavior at the onset of optic cup formation.

**Figure 4:**
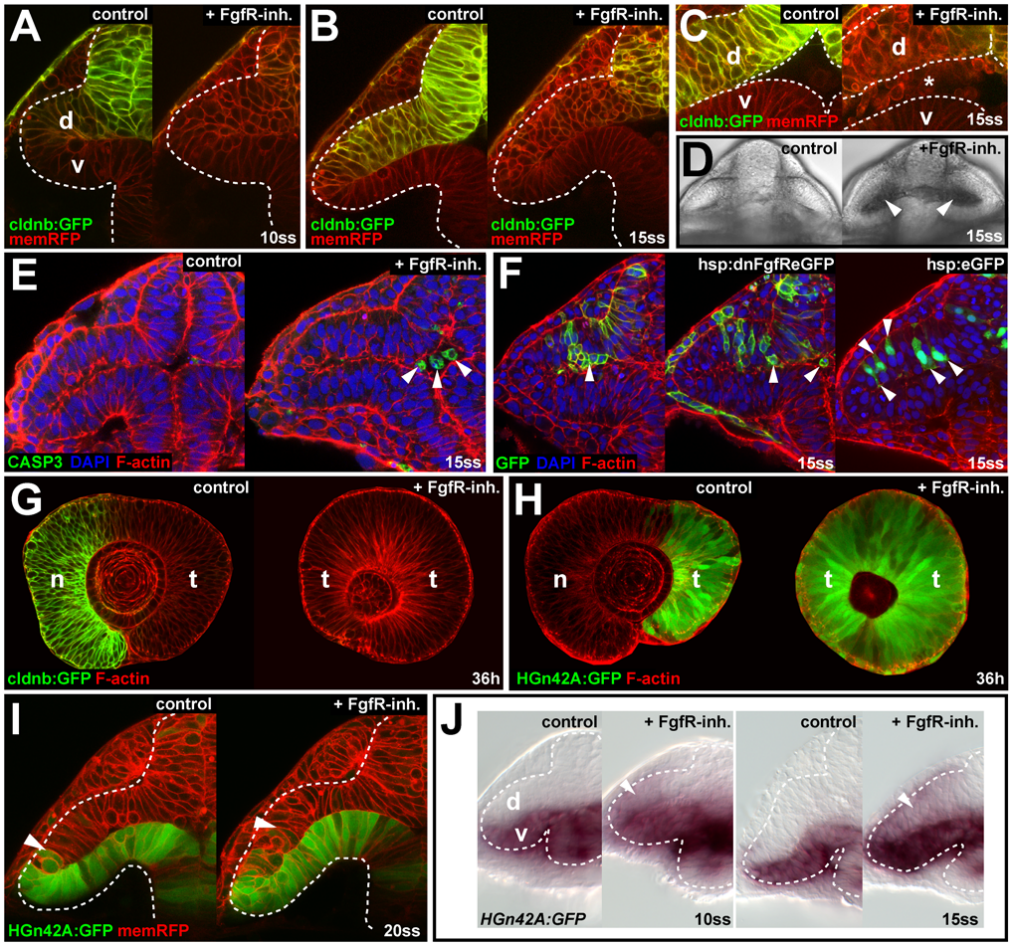
Fgf signaling is required for epithelial integrity of nasal retina progenitors. (A–D) Live images of wt control (left) and FgfR-inh.-treated embryos (right). *Tg(-8.0cldnb:lynGFP)zf106* (green) and memRFP colabel (red) at 10ss, 2 h after treatment (hpt) (A), at 15ss, 4 h hpt at low magnification (B), high magnification (C), and in bright field (D) (asterisk in [C] and arrowheads in [D]: delaminated cells in the optic vesicle ventricle). (E) Apoptosis detection with anti-CASP3 (green), counterstained for cortical F-actin (red) and DAPI (blue) in wt control (left) and FgfR-inh.-treated embryo (right) at 15ss, 4 hpt (arrowheads: delaminated cells in the optic vesicle ventricle). (F) Single cells in *Tg(hsp70l:dnfgfr1-EGFP)pd1* clones (left two panels) aggregate (left) and protrude apically and are found in the ventricle (middle) at 15ss, 5 h after heat shock, compared to identically treated *hsp70l:eGFP* control clones (right). Counterstaining: F-actin (red), DAPI (blue). (G) cldnb:GFP expression (green), counterstained for F-actin (red) in the nasal retina of control (left) is absent after FgfR-inh. treatment (right) at 36 h (single, longitudinal confocal sections with nasal to the left). (H) HGn42A:GFP expression (green), counterstained for F-actin (red) in the temporal retina of control (left) is expanded throughout the retina after FgfR-inh. treatment (right) at 36 h (single, longitudinal confocal sections with nasal to the left). (I) Live images of HGn42A:GFP expression (green) in wt control (left) and FgfR-inh.-treated embryos (right) at 20ss, colabeled with membrane-targeted RFP (red) (arrowheads: distal GFP expression limit). (J) HGn42A:GFP transcription detected by GFP in situ hybridization at 10- (left) and 15ss (right) in the ventral optic vesicle leaflet of control embryos is expanded into the dorsal optic vesicle leaflet upon FgfR-inh. treatment (white arrowheads). Orientation in (A–F, I, and J): cross-sections through one half of the forebrain, dorsal to the top and lateral to the left, in (G and H): lateral with nasal/anterior to the left and dorsal to the top. Dotted lines: neural tube boundary. d, dorsal optic vesicle leaflet; n, nasal; t, temporal; v, ventral optic vesicle leaflet.

Increasingly reduced levels of cldnb:GFP expression in the dorsal optic vesicle leaflet after FgfR-inh.–treatment indicates that reporter expression depends on Fgf signaling (Figure 4A and 4B; *n* = 7/7). At 15ss, cells in the dorsal leaflet appear disorganized, whereas cells in the lower leaflet appear normal (Figure 4B), the tight apical membrane apposition of the optic vesicle leaflets is lost, and the ventricle contains delaminated, weakly cldnb:GFP-positive (unpublished data) cells (Figure 4C and 4D, *n* = 5/5). The delaminated cells eventually undergo apoptosis, but viability of the disorganized cells in the optic vesicle neuroepithelium is not compromised (Figure 4E). To test whether an early defect in apical-basal cell polarity causes later delamination, we studied the expression of the two apical markers aPKC and ZO1. At 10ss, prior to cell delamination, expression of both markers is normal in FgfR-inh.–treated embryos compared to controls (Figure S8A and S8B). At 15ss, after the onset of delamination, expression reflects the loss of apical membrane apposition and accumulation of cells in the optic vesicle ventricle of FgfR-inh.–treated embryos, but the general apical-basal polarity of the optic vesicle epithelium is not affected (Figure S8C and S8D and unpublished data).

To directly assess the requirement for FgfR-signaling in nasal retina progenitors, we transplanted *Tg(hsp70l:dnfgfr1-EGFP)pd1*–positive cells [34] that express a dominant-negative, GFP-tagged version of Fgfr1 under the control of a heat-shock promoter, into wt host embryos and heat-shocked the chimeras at the onset of optic vesicle evagination. At 12ss, embryos with *dnfgfr1*-EGFP clones in the dorsal optic vesicle leaflet show reduced *foxg1* (*n* = 10/12) and ectopic expression of *foxd1* (*n* = 8/12) (Figure S9), reminiscent of *fgf8/3/24^−/−^* embryos (Figure 1E and 1F, right). Many abnormally cuboidal *dnfgfr1*-EGFP–expressing cells in the dorsal optic vesicle leaflet accumulate at the apical side of the neuroepithelium and often protrude and delaminate into the ventricle at 15ss (Figure 4F, *n* = 7/8, left and middle), similar to the effect of the FgfR-inh. Cell and epithelial morphology in control chimeras that express eGFP under the control of the heat-shock promoter is normal (Figure 4F, *n* = 9/9, right). Thus, misspecification of the dorsal optic vesicle leaflet in the absence of Fgfs—now *foxg1*-negative—leads to a defect in neuroepithelial integrity and subsequent loss of presumptive nasal retina progenitors. Consistent with a loss rather than a temporal misspecification of nasal progenitors, there is no perdurance of nasal cldnb:GFP expression in the retinae of FgfR-inh.–treated embryos at 36 h (Figure 4G; *n* = 8/8). The complementary expansion of temporal HGn42A:GFP expression in retinae of FgfR-inh.–treated embryos at 36 h indicates a complete loss of nasal cell fates (Figure 4H).

Although the behavior of prospective nasal cells is disrupted upon abrogation of Fgf signaling, an optic cup still forms. To explore how eye morphogenesis occurs in such circumstances, we followed the movements of HGn42A:GFP-labeled temporal progenitors by in vivo imaging in FgfR-inh.–treated embryos. GFP mRNA in HGn42A embryos is ectopically found in the dorsal optic vesicle leaflet after FgfR-inh. treatment, similar to the effect on *foxd1*, but GFP protein maturation appears to lag behind, therefore allowing the tracking of ventral optic leaflet cells (Figure 4J). At 20ss, more HGn42A:GFP-positive temporal progenitors have moved into the future neural retina domain of the outer optic cup layer in FgfR-inh.–treated embryos compared to controls (Figure 4I; *n* = 7/7). This suggests that the loss of misspecified *foxg1*-negative, nasal progenitors in the optic cup after FgfR inhibition causes an enhanced movement of temporal progenitors from the ventral leaflet into the future neural retina domain.

### Temporal Progenitor Cell Movement Can Occur Independent of Fgf-Mediated Patterning

**Figure 5:**
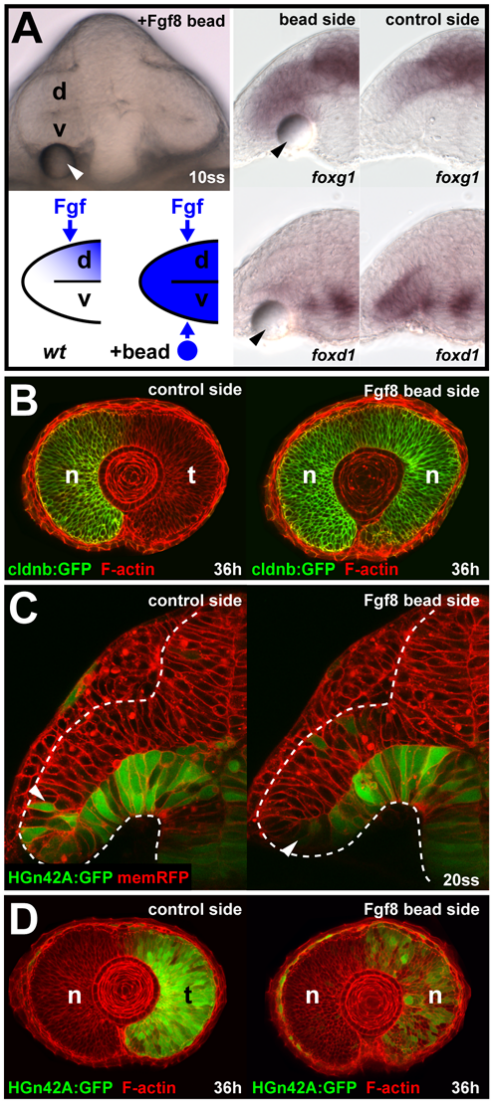
Cell movements into the neural retina can occur independent of Fgf-dependent nasal-temporal patterning. (A) Live embryo at 10ss, 3 h after Fgf8 bead implantation (arrowhead) below the ventral optic vesicle leaflet (top left). Predicted Fgf distribution along the dorsal-ventral axis of the optic vesicle in wt control and after Fgf8 bead implantation (bottom left). Ectopic *foxg1* expression (top) and repression of *foxd1* (bottom) in the ventral optic vesicle at 10ss, after Fgf8 bead implantation (left) compared to the control side of the same embryo (right). (B) Nasal cldnb:GFP expression (green) at 36 h on the control side (left) and the *double-nasal* Fgf8 bead implantation side (right) of the same embryo (red: F-actin counterstain). (C) Live images of HGn42A:GFP expression (green), colabeled with membrane-targeted RFP (red) at 20ss after Fgf8 bead implantation (left: control side, right: bead implantation side, arrowheads: distal GFP expression limit). Dotted lines: neural tube boundary. (D) Temporal HGn42A:GFP expression (green) at 36 h on the control side (left) and the *double-nasal* Fgf8 bead implantation side (right) of the same embryo (red: F-actin counterstain). Orientation in (A and C): cross-sections, dorsal to the top and lateral to the left; in (B and D): lateral with nasal/anterior to the left and dorsal to the top. d, dorsal optic vesicle leaflet; n, nasal; t, temporal; v, ventral optic vesicle leaflet.

To complement the analysis of morphogenetic movements in eyes lacking nasal identity, we created eyes that lack temporal identity by implanting Fgf8 beads adjacent to the nascent temporal retina, below the ventral optic vesicle leaflet (Figure 5A and 5B). The movement of HGn42A:GFP-positive cells from the ventral optic vesicle leaflet into the outer optic cup layer is delayed after Fgf8 bead implantation compared to the control side of the same embryo (Figure 5C; *n* = 8/9). However, perdurance of HGn42A:GFP expression shows that these cells eventually reach their normal axial position in the retina at 36 h (Figure 5D; *n* = 12/12). Thus, the morphogenetic movement of temporal retina progenitor cells from the ventral optic vesicle leaflet around the distal ridge into the prospective neural retina can occur independent of correctly restricted *foxg1* and *foxd1* expression.

### Foxg1 Is Required for Epithelial Cell Cohesion in the Optic Vesicle

The dependence of *foxg1* expression upon Fgf signaling raised the possibility that Foxg1 may be a transcriptional mediator of some or all of the effects of Fgf signaling upon presumptive nasal progenitors. To test this idea, we studied *foxg1* in loss- and gain-of-function assays.

**Figure 6:**
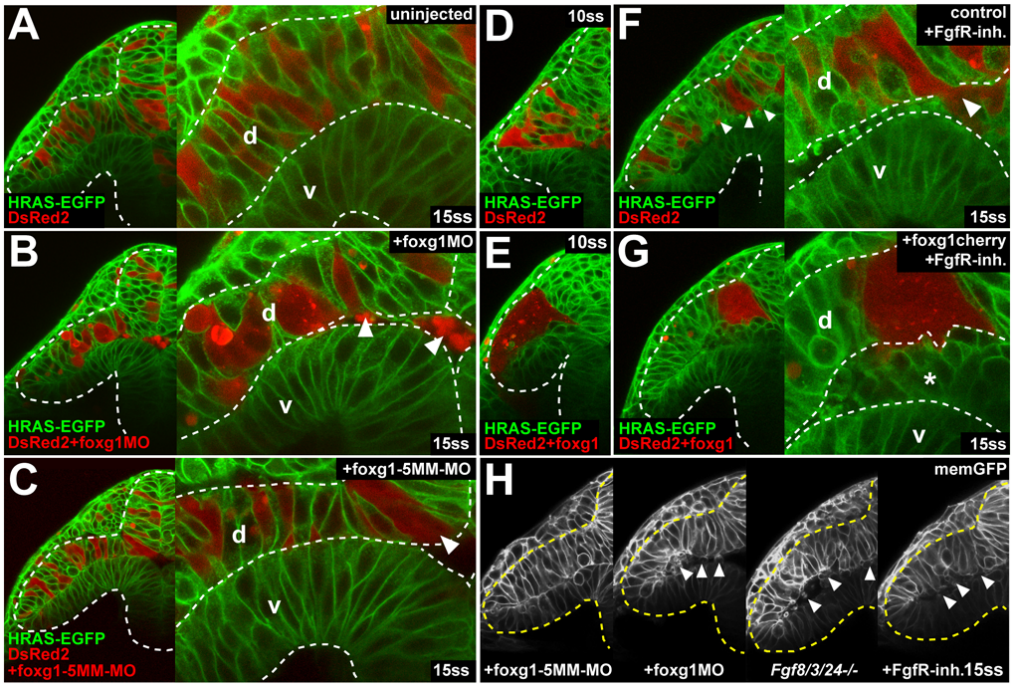
Foxg1 is required for cell cohesion in the optic vesicle. (A–C) Live images of a DsRed2-expressing cell clone from a noninjected control donor (A), a foxg1-morpholino–injected donor (B), and a foxg1-mismatch-control-morpholino–injected donor in a *Tg(Bactin:HRAS-EGFP)vu119* host embryo (C) at 15ss (right: high magnification view of the dorsal and ventral leaflet in the region of the proximal optic vesicle, arrowheads: delaminating cells). (D and E) Live images of a DsRed2-expressing cell clone from a noninjected control donor (D) and a foxg1-RNA–injected donor (E) in a *Tg(Bactin:HRAS-EGFP)vu119* host embryo at 10ss. (F and G) Live images of a DsRed2-expressing cell clone from a noninjected control donor (F) and a foxg1-injected donor (G) in a *Tg(Bactin:HRAS-EGFP)vu119* host embryo in the presence of FgfR-inhibitor at 15ss (right: high magnification view of the dorsal and ventral leaflet in the region of the proximal optic vesicle, arrowheads in [F] and asterisk in [G]: delaminating cells). (H) Live images of cell delamination (arrowheads) in a foxg1-mismatch-control-morpholino–injected embryo, a foxg1-morpholino–injected embryo, an *fgf8/3/24^−/−^* embryo, and an embryo after FgfR.-inh. treatment at the 15ss stage, coinjected with memGFP RNA (from left to right). Orientation: cross-sections, dorsal to the top and lateral to the left. Dotted lines: neural tube boundary. d, dorsal optic vesicle leaflet; v, ventral optic vesicle leaflet.

Live imaging of optic vesicles in memGFP-labeled embryos, shows that abrogation of Foxg1 using a translation-blocking morpholino (foxg1MO; Figure S10) results in the delamination and accumulation of cells in the ventricle of the optic vesicle at 15ss (*n* = 7/8), when compared to controls injected with a 5-bp mismatch control morpholino (foxg1-5MM-MO, *n* = 8/8) (Figure 6H, left two panels). This phenotype is highly similar to the delamination observed in *fgf8/3/24^−/−^* embryos and after FgfR inhibition (Figure 6H, right two panels). Except for this phenotype and the previously reported changes during telencephalic development [35], this foxg1 morpholino does not create any morphological defects (unpublished data), consistent with its specificity and the restriction of *foxg1* expression to the developing forebrain at this stage. Live imaging reveals that only foxg1MO-injected DsRed2 donor cells delaminate when transplanted into noninjected HRAS-GFP–expressing hosts (*n* = 9/10); neither host cells nor DsRed2-positive control transplanted cells (*n* = 10/10), nor foxg1-5MM-MO–injected cells show this phenotype (Figure 6A–6C). Next, we performed clonal overexpression by transplanting foxg1cherry-injected DsRed2 donor cells into noninjected HRAS-EGFP hosts. Compared to clones of noninjected cells, which scatter by mixing with host cells, foxg1cherry-overexpressing cells form highly coherent clusters, with hardly any host cells intermingling at 10ss (Figure 6D and 6E; *n* = 15/15). Together, these results suggested that Foxg1 may act downstream of Fgf signaling to promote epithelial cohesion of cells in the nascent nasal retina.

Supporting this hypothesis, we found that Foxg1 could rescue the delamination of nasal retinal cells that occurs upon abrogation of Fgf signaling. In chimeras in which Fgf signaling is blocked, foxg1cherry-overexpressing cells are rescued from delamination (*n* = 12/12), when compared to host cells and noninjected transplanted cells (Figure 6F and 6G; *n* = 9/11). This strongly suggests that Foxg1 promotes cell cohesion in the dorsal optic vesicle leaflet, and that the delamination and death of misspecified nasal progenitors observed in the absence of Fgf signaling is due to the lack of Foxg1.

### Foxg1 Promotes Cell Clustering at Sites of High Fgf-Signaling Activity

In chimeras carrying foxg1-overexpressing cells, we observed that the large majority of coherent clones were found in the dorsal forebrain, dorsal optic vesicle leaflet, head mesenchyme surrounding the optic vesicle, and olfactory primordium (unpublished data), all sites of high Fgf pathway activity. To explore this phenomenon, we tracked the lateral spreading/clustering of foxg1-overexpressing cell clones in response to exogenous Fgf provided from a bead.

**Figure 7:**
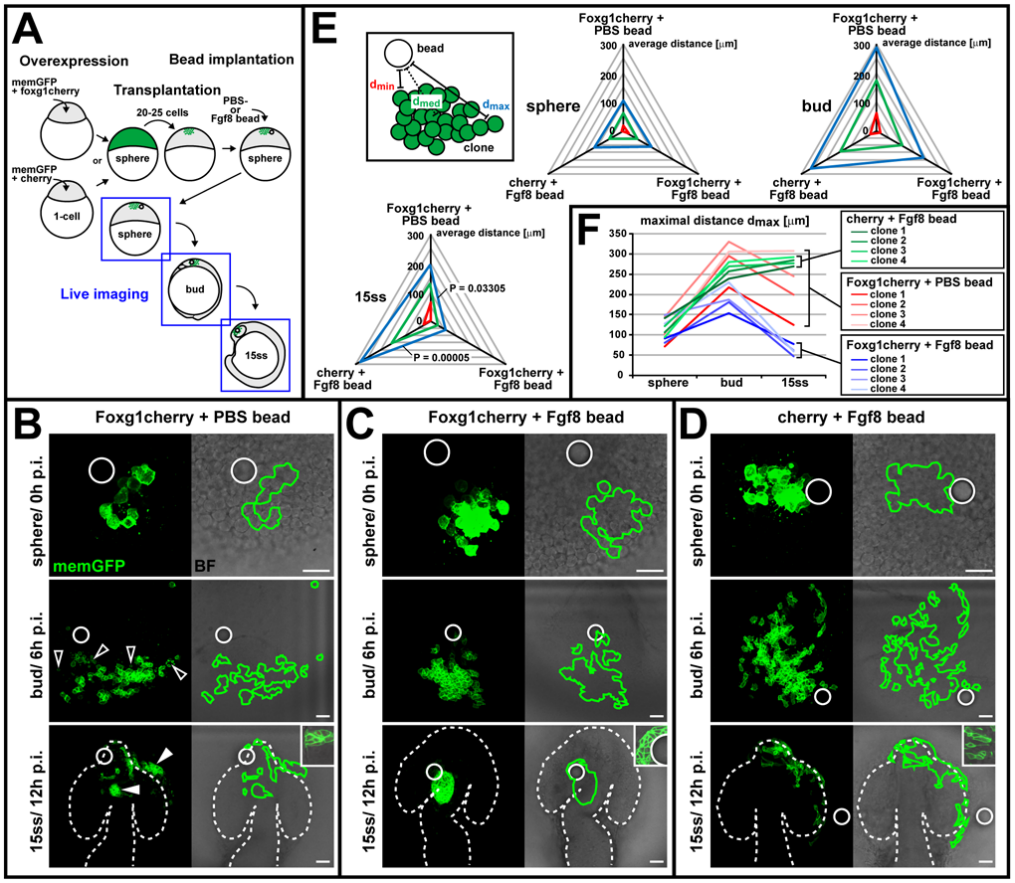
Foxg1 mediates cell cohesion in an Fgf-dependent manner. (A) Schematic of the *overexpression-cell-transplantation-bead-implantation* assay to test for an Fgf-dependent effect of *foxg1* on cell cohesion by monitoring lateral cell spreading in live embryos. (B–D) Live images of horizontal cell spreading at sphere (0 h postimplantation [p.i.], top), bud (6 h postimplantation, middle), and 15ss (12 h postimplantation, bottom) from a representative clone overexpressing foxg1cherry in the presence of a PBS control bead (B), a clone overexpressing foxg1cherry in the presence of an Fgf8 bead (C), and a clone overexpressing cherry in the presence of an Fgf8 bead (D). Left: memGFP image (confocal *z*-projection), right: clone outline (green line), derived from memGFP image, superimposed with a bright-field (BF) image, showing bead localization (white circle). Dotted outlines: neural tube border. Insets in lower panels show cell morphology (bright-field image superimposed with a single confocal section of memGFP). Open arrowheads in (B): abnormal cells not included in tracking the clone outline. White arrowheads in (B): autonomous clustering of foxg1-overexpressing cells in the olfactory placode, head mesenchyme, and telencephalon. Scale bar: 50 µm. (E) Quantification of lateral cell spreading relative to the site of bead implantation. The maximal cell distance (*d*_max_, blue) and the minimal distance from the bead surface (*d*_min_, red) was measured (on projected *z*-stacks) and the median distance (*d*_med_, green) calculated on the basis of *d*_max_ and *d*_min_ for four representative clones at sphere, bud, and 15ss. Radial plots of average *d*_max_, *d*_min_, and *d*_med_ values (in micrometers) at sphere, bud, and 15ss, (*n* = 4/stage) for foxg1cherry overexpression in the presence of a PBS control bead (top axis), foxg1cherry overexpression in the presence of an Fgf8 bead (right axis), and cherry overexpression in the presence of an Fgf8 bead (left axis). *p*-Values for 15ss stage derived by a paired, two-tailed *t*-test. (F) Line plots of single *d*_max_ values (in micrometers) over time (sphere, bud, 15ss) for four representative clones overexpressing foxg1cherry in the presence of a PBS control bead (red), overexpressing foxg1cherry in the presence of a Fgf8 bead (blue), and overexpressing cherry in the presence of a Fgf8 bead (green). Brackets indicate *d*_max_ value range at 15ss.

For this purpose, first, 20–25 cells from memGFP-labeled donors, either overexpressing foxg1cherry or cherry (control) protein, were transplanted into nonlabeled hosts at sphere stage. Directly afterwards, beads, either coated with recombinant Fgf8 protein or PBS (control), were implanted next to the transplanted cell clone. Individual bead-implanted chimeras were then separately analyzed by live imaging at sphere (directly after implantation), bud (6 h after implantation), and 15ss (12 h after implantation) (Figure 7A).

Directly after implantation (sphere), spreading of foxg1cherry-overexpressing clones in the presence of a PBS bead (Figure 7B, top), foxg1cherry-overexpressing cells in the presence of an Fgf8 bead (Figure 7C, top) and cherry-overexpressing cells in the presence of an Fgf8 bead (Figure 7D, top) is very similar, when comparing the average of the measured maximal (*d*_max_), measured minimal (*d*_min_), and calculated median (*d*_med_) distance of cells to the bead surface from four representative experiments for each condition (Figure 7E, first radial plot). At bud, live imaging and measuring *d*_max_ (Figure 7E, second radial plot) of foxg1cherry-overexpressing cells in the presence of an Fgf8 bead (Figure 7C, middle) show slightly decreased spreading, when compared to *foxg1* overexpression with a PBS bead (Figure 7B, middle) or cherry overexpression with an Fgf8 bead (Figure 7D, middle). At 15ss, all analyzed foxg1cherry-overexpressing clones in the presence of an Fgf8 bead formed a tightly aggregated, single cluster at the site of bead implantation (Figure 7C, bottom). Foxg1cherry-overexpressing clones in the presence of a PBS bead form several scattered clusters that do not coincide with the bead implantation site (Figure 7B, bottom), and Fgf8 beads do not induce clustering of cherry-overexpressing clones (Figure 7D, bottom). Analysis of *d*_max_ at 15ss revealed a significant reduction for foxg1cherry-overexpressing clones in the presence of an Fgf8 bead compared to foxg1cherry-overexpressing clones in the presence of a PBS bead (*p* = 0.03305) or cherry-overexpressing clones in the presence of an Fgf8 bead (*p* = 0.00005) (Figure 7E, third radial plot). Plotting *d*_max_ over time shows that the spreading behavior of foxg1cherry-overexpressing clones in the presence of an Fgf8 bead diverges from bud stage onwards when compared to cherry-overexpressing cells in the presence of an Fgf8 bead. At 15ss, *d*_max_ of foxg1cherry-overexpressing cells in the presence of a PBS bead shows a high degree of variability between individual clones, probably depending on their relative location to endogenous Fgf sources (Figure 7F).

These results show that *foxg1*-expressing cells preferentially cluster around Fgf sources, suggesting a positive feedback between Fgf-dependent regulation of *foxg1* gene expression and sustained cohesion of *foxg1*-expressing cells close to Fgf sources.

## Discussion

### Stages and Dynamics of Nasal-Temporal Axis Development

Experiments in chick embryos have long suggested specification of the nasal-temporal axis of the retina at early stages of eye morphogenesis [36], and our previous work indicated that nasal-temporal axis formation requires early Fgf8 signaling [25]. We now show that a combined Fgf8/3/24 signal along the dorsal-ventral axis of the neural tube fully controls the nasal-temporal subdivision of the future neural retina already at the onset of optic vesicle evagination.

This unexpected, early orientation of the future nasal-temporal axis raises the question of how its final alignment with the anterior-posterior body axis is achieved. After the onset of evagination, the optic vesicle enters a phase of growth and lateral extension [37]. We show that during this process, the dorsal leaflet of the optic vesicle, which is facing the ectoderm, consists only of nasal retinal progenitors. Subsequently, the optic vesicle is transformed into the two-layered optic cup. At this stage, nasal retina progenitors in the outer layer gradually regress into their final axial position by epithelial compaction, concordant with late movement of temporal retina progenitors from the inner layer, around the distal ridge of the optic cup. The synchronization of these morphogenetic processes is crucial for the final nasal-temporal subdivision of the neural retina and could explain why fate-mapping experiments previously revealed an alignment of nasal-temporal cell positions along the medial-lateral axis of the optic cup [19].

**Figure 8:**
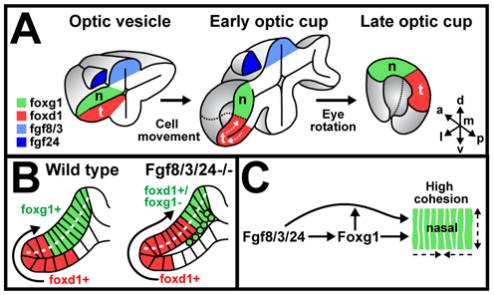
Model for retinal patterning and morphogenesis along the nasal-temporal axis. (A) Schematic cross-section, illustrating nasal-temporal pattern formation during optic vesicle and optic cup morphogenesis. Fgf8/3/24-dependent axis specification occurs along the dorsal-ventral axis of the optic vesicle (left). Initially, only nasal retina progenitors (green) populate the future neural retina domain. During optic cup formation, temporal progenitor cell movements (red) lead to axis reorientation (middle). In parallel, the eye rotates anteriorly. Together, these movements result in the anterior-posterior alignment of the nasal-temporal retina axis (right). (B) Directed, late movement of *foxd1*-expressing, temporal progenitors (red) around the distal ridge into the outer optic cup layer and gradual displacement of *foxg1*-expressing nasal progenitors (green) leads to the final nasal-temporal subdivision of the definitive neural retina domain in the outer optic cup layer (white dotted line). In the absence of Fgf8/3/24, misspecified (*foxg1*-negative/*foxd1*-positive) retinal progenitors in the presumptive nasal region (green) delaminate, and temporal progenitors (red) show increased cell movements into the outer optic cup layer. (C) Effect of Fgf signaling on cell cohesion and morphogenesis. Fgf8/3/24 are first required for *foxg1* gene expression in nasal retina progenitors. Foxg1 protein enhances cell cohesion in the presence of Fgfs through an unknown feed-forward mechanism. The resulting retention of Foxg1-expressing cells at sites of Fgf signaling enhances the feed-forward effect. a, anterior; d, dorsal; l, lateral; m, medial; n, nasal; olf, olfactory placode; p, posterior; t, temporal; v, ventral.

Thus, formation of the nasal-temporal axis occurs as the results of three processes: (1) axis specification in the optic vesicle along the dorsal-ventral axis of neural tube, (2) morphogenetic axis reorientation by cell morphology changes and directed cell movements, and (3) 90° anterior rotation of the optic cup [37], leading to the alignment with the anterior-posterior body axis (Figure 8A).

### Fgf Spreading and Competence during Pattern Formation in the Optic Vesicle

All three Fgfs involved in nasal-temporal pattern formation originate asymmetrically relative to the future axis, consistent with their role in nasal fate specification in the dorsal optic vesicle leaflet. *Fgf8* and *-3* are both expressed in the dorsal forebrain [38],[39] and *fgf24* in cells of the olfactory placode [26],[40].

The graded transformation of nasal into temporal retina fates upon stepwise elimination of *fgf8/3/24* and the nested expression of Fgf target genes in the optic vesicle, suggest a morphogen-like mechanism with continuous determination of cell positional identities along an Fgf gradient. This would be similar to the mechanism of action of Fgf signaling during mesoderm development [41]. Such a gradient could arise by propagation of Fgf8 and Fgf3 within the neuroepithelium, since early, the optic vesicle is contiguous with the dorsal forebrain. In contrast, the source of Fgf24 lies outside the neural tube, and it thus must signal vertically through the basal side of the optic vesicle neuroepithelium. Bead implantations into the tissue surrounding the optic vesicle indicate that Fgf8 can also signal effectively via the basal side of the neuroepithelium. Interestingly, Fgf signaling occurs via the basal side of the epithelium during otic vesicle invagination [42]. We thus favor the model of an extra-neuroepithelial gradient of Fgf8/3/24 during optic vesicle patterning.

Bead implants show that the ventral optic vesicle leaflet—the future temporal retina—is also competent to respond to Fgfs. The spatially restricted response to Fgfs in the dorsal optic vesicle leaflet could, therefore, occur by limiting signal spreading. One possibility is that the tight apposition between cells of the pre-lens ectoderm and the distal optic vesicle ridge prevents Fgf spreading to ventral cells, thereby leading to a sharp signaling threshold that underlies the precise dorsal-ventral restriction of *foxg1* and *foxd1* expression in the optic vesicle.

Recently, Fgf19 has been suggested to act during lens and retina development, possibly downstream of Fgf8 and Fgf3 [43]. Fgf19 loss- and gain-of-function experiments can alter retinal gene expression levels, but fail to produce any consistent nasal-temporal patterning shift independent of severe abnormalities in optic cup morphology. Together with its unrestricted and late expression, this makes a specific contribution of Fgf19 to nasal-temporal patterning very unlikely. The previously reported cooperation of Fgf8 and Fgf3 during retinal neurogenesis [44] appears to be unrelated to the function of Fgf8/3/24 during early pattern formation and eye morphogenesis (unpublished data).

### Roles of Foxg1 in Pattern Formation and Neural Progenitor Proliferation

Experiments in mouse and chick embryos have shown a mutually repressive interaction between *foxg1* and *foxd1* [10],[45]. This is supported by the sharp *foxg1*/*-d1* expression boundary at the distal ridge of the optic vesicle. Additionally, we find that *foxg1* and *foxd1* expression always occurs in a mutually exclusive pattern in both Fgf loss- and gain-of-function experiments. Similar to the mouse telencephalon [46],[47], *foxg1* in the optic vesicle may be directly activated by Fgf signaling. Since Fgf signaling only regulates the development of nasal fates, temporal fate specification and *foxd1* expression in the ventral optic vesicle leaflet could either occur as default, in the absence of Fgf signals, and/or as response to a different signal, potentially of ventral origin.

*Foxg1* and *-d1* are likely to act as direct regulators of *efn*/*eph* guidance cue expression [10],[11]. Although Efna5a and Epha4b are guidance cues for retinal ganglion cell axons, we show that their spatially restricted expression is already established in the optic vesicle, shortly after that of *foxg1* and *foxd1* and long before neuronal differentiation and retinotectal map formation. Given that Efns/Ephs are known effectors of cell sorting at lineage-restricted compartment boundaries [48], a potential, early role for Efn/Eph signaling could be maintenance of a nasal-temporal lineage boundary between cells in the dorsal and ventral leaflets of the optic vesicle during growth and morphogenesis. Indeed, the presence of such lineage-restricted compartments along the dorsal-ventral axis of the retina has previously been suggested by fate mapping experiments in chick embryos [49].

Foxg1 is best studied for its function in maintaining neural progenitor proliferation [27]–[29]. We find that the optic vesicle contains 2-fold more mitotic, *foxg1*-positive, nasal retina progenitors than mitotic, *foxd1*-positive, temporal retina progenitors. This asymmetry depends on Fgf signaling and coincides with the strict Fgf requirement for *foxg1* expression. One role for *foxg1* could thus be maintenance of a high rate of neural progenitor proliferation, close to the source of Fgfs, similar to the role proposed for Fgfs in the vertebrate spinal cord stem zone [50].

### Epithelial Cohesion and Cell Movements during Optic Cup Morphogenesis

Changes in motility and/or shape of individual cells must be tightly balanced with cell–cell adhesion to assure tissue integrity during epithelial morphogenesis, but how positional identities defined during pattern formation contribute to regional differences in morphogenetic cell behavior is poorly understood [51],[52]. We find that local cell behavior and epithelial integrity during optic cup morphogenesis directly depend on correct prior patterning of the optic vesicle by Fgfs.

When *foxd1*-expressing, temporal progenitors move into the optic cup, *foxg1*-expressing, nasal progenitors increasingly regress dorsoproximally, leading to the definitive alignment of nasal-temporal fates in the primordium of the neural retina (Figure 8B, left). In the course of this morphogenetic movement, nasal progenitors appear increasingly immotile and elongated, whereas temporal progenitors in the inner optic cup layer appear motile and more cuboidal. There are two plausible, but not exclusive, explanations for the driving force behind the morphogenetic cell movement and cell shape changes in the optic cup: on one hand, an active movement of temporal cells could exert a force within the plane of the optic cup neuroepithelium, leading to the gradual compaction of nasal progenitors already residing in the future neural retina domain (*pushing force*). Fgf8 bead implants indicate that the movement of cells from the ventral optic leaflet into the neural retina can occur independent of their Fgf-dependent nasal-temporal fate. Thus, if this movement is active, it appears to be under the control of a yet unidentified factor.

Alternatively, since the optic cup epithelium is overall highly coherent, the compaction of future nasal tissue could *tow* temporal progenitors into the neural retina, independent of active, lateral cell movements (*pulling force*). The higher rate of temporal progenitor movement observed in the absence of Fgf signaling suggests that intact cohesion of nasal progenitors at least restricts morphogenetic movements of temporal cells. Evidently, compaction by cell elongation can only occur if the nasal progenitors, already residing in the future neural retina domain, have a high degree of lateral cohesion. In support of this, we find that in the absence of Fgf signaling and Foxg1 activity, nasal progenitors start to delaminate exactly at the onset of temporal progenitor movement into the optic cup (Figure 8B, right). Together with the ability of Foxg1 to rescue delamination in the absence of Fgf signaling and the strong effect of Foxg1 overexpression on lateral cell spreading/clustering, this suggests a novel role for Foxg1 as a positive regulator of neuroepithelial cell cohesion.

When Foxg1 is ectopically expressed, cell cohesion is more pronounced in regions of high Fgf signaling activity. There could be several explanations for this. First, Fgf signaling might enhance Foxg1-dependent *foxg1* expression, translation or posttranslational efficacy, such that Foxg1 function is more effective in an Fgf-signaling environment. Indeed, it has been recently shown that Fgf signaling posttranslationally regulates the subcellular localization of Foxg1 [53], indicating a role for persistent Fgf signaling on Foxg1 function. Furthermore, there could be a feed-forward mechanism whereby Foxg1 enhances an independent role for Fgf signaling in mediating cell cohesion (Figure 8C). In support of this, recent results suggest a requirement for Fgf signaling in epithelial organization during collective cell migration in the lateral line primordium [54],[55] and perhaps to a lesser extent in the parapineal nucleus [56]. In the lateral line primordium, Fgf signaling may promote epithelialization and the formation of apical junctional complexes between the polarized epithelial cells [54]. A failure in maintaining neuroepithelial integrity and junctional complexes could certainly contribute to the observed extrusion of prospective nasal cells in the absence of Fgf signaling. Additionally, chemotactic effects of Fgfs [57] could limit nasal progenitor spreading and thereby indirectly lead to epithelial compaction. The future identification of Fgf- and Foxg1-regulated effectors of cell adhesion will advance the understanding of this mechanism. The phenotype of *fgf8/3/24^−/−^* embryos is reminiscent of the general loss of neuroepithelial integrity in N-cadherin mutants [58],[59]. It is thus possible that Fgf-dependent *foxg1* expression is locally required for cadherin-mediated cell adhesion. However, a disruption of apical-basal cell polarity does not appear to be the cause of cell delamination in *fgf8/3/24^−/−^*.

In summary, by in vivo tracking, the development of one retinal axis from its specification, through morphogenetic rearrangement until its final orientation, we show that retinal pattern formation and morphogenesis are tightly coordinated processes. Considering the differences of optic vesicle morphology between vertebrate species [60],[61], it will be important to assess the conservation of morphogenetic axis reorientation. In first support of this, a recent fate-mapping study suggests that nasal-temporal cell positions initially align along the dorsal-ventral neural tube axis also in chick embryos [62]. Interestingly, in frog embryos, late cell movement from the optic stalk contributes to formation of the ventral neural retina [63], suggesting comparable cell movements may shape the retina along both of its major axes.

## Materials and Methods

### Fish Lines and Maintenance

Fish were maintained and bred according to standard procedures [64]. AB or tupl wild-type and *ace^ti282a^* (*acerebellar/fgf8*), *ika^t22030^* (*ikarus/fgf24*), and *fgf3^t24149^* mutant fish were used for intercrossing and to breed [38],[65],[66]. Adult carrier fish and mutant embryos were identified by direct sequencing after PCR on genomic DNA, using the following primers: Fgf3-forward: 5′-TCTTCAACCGAGAGTGTGAGTTTCTA-3′, Fgf3-reverse: 5′-CGCTGACTCTCTCTAAGCTTGCGC-3′, Fgf8-forward: 5′-AGACGGACACATTTGGGAGTCGAGT-3′, Fgf8-reverse: 5′- AAGTCACAAAAGTGATGACTTTTTCAGATA-3′, Fgf24-forward: 5′- TTGTATTTTGCAGCTCTGCTTGTGGTC-3′, Fgf24-reverse: 5′- TGTGGCTGTGTCCAGATGTTGTACG-3′. The following transgenic lines were used: *Tg(-8.0cldnb:lynGFP)zf106*, *HGn42A*, *Tg(hsp70l:dnfgfr1-EGFP)pd1*, *Tg(h2afv:GFP)kca66*, *Tg(Bactin:HRAS-EGFP)vu119* [31]–[34],[67], and a line expressing DsRed2 under the control of the *Xenopus ef1a* promoter [68]. The *cldnb* gene is not expressed in the retina, and nasal retina expression is only present in the single *Tg(-8.0cldnb:lynGFP)zf106* line (D. Gilmour, personal communication), thus representing a positional effect. The nature of the trapped enhancer is currently unknown.

### In Situ Hybridization, Fgf24 Morpholino Injection, FgfR Inhibitor Treatment, and Fgf8 Bead Implants

Whole-mount mRNA in situ hybridizations were done as described [38]. Triple inactivation of *fgf8*, *-3*, and *-24* was achieved by injecting an *fgf24* antisense morpholino [26] (MO) into fertilized eggs from *fgf8/3* double-mutant carrier crosses. The *fgf24*-MO was titrated to 1 nl/embryo of a 0.2 mM MO solution in 1× Danieau's medium with 0.02 mg/ml Fast Green FCF (Fluka) by comparing *fgf24*MO-injected, *fgf8* mutant embryos with the phenotype of genetic *fgf8/24* double mutants. FgfR-inhibitor treatment with 5 µM SU5402 (Calbiochem) in E3 medium was done between the 1- and 5ss stages on dechorionated embryos in agarose-coated 24-well plates. Controls were treated with 0.05% DMSO in E3. Polystyrene beads (40 µm; Polysciences) were loaded with 250 µg/ml recombinant zebrafish Fgf8 (A. Picker, unpublished data) or recombinant mouse Fgf8b (R&D Systems) and implanted as previously described [25].

### Foxg1 Morpholinos and Overexpression

Two *foxg1* antisense morpholinos generated the same cell delamination phenotype: foxg1-MO1 (5′-CTTTTCTTTCTCCCATATCCAACAT-3′ [35]) and foxg1-MO2 (5′-CCCATATCCAACATCACAAGTAAG-3′) [69] As control for foxg1-MO1, a 5-mismatch morpholino was used (5′- CTaTTgTTTCTCgCATATgCAAgAT-3′). All foxg1 morpholinos were injected at: 0.5–1 nl of MO/embryo (1 mM). For foxg1 overexpression, embryos were injected with 0.5 nl of in vitro–transcribed RNA (100 ng/µl) encoding Foxg1cherry. The specificity of translation blocking by foxg1-MO1 was tested by coinjection with foxg1cherry RNA into *Tg(h2afv:GFP)kca66* embryos which ubiquitously express GFP-tagged histone 2a.

### Cell Transplantation and Heat-Shock Treatment

All cell transplantations were carried out between the 40% and 50% epiboly stages at the animal pole. *Dnfgfr1*-expressing cell clones were created by transplantation of cells from *Tg(hsp70l:dnfgfr1-EGFP)pd1* embryos into the animal pole of wt embryos at late blastula stages. Chimeras were heat-shock induced by transfer into 37°C E3 medium at the 3ss stage and subsequently incubated at 28.5°C. Controls chimeras carrying clones from a *hsp70l:eGFP* transgenic line (S. Hans, unpublished data) were treated identically.

### Qualitative Profiling of Gene Expression

Sets of 8-bit grayscale images of dissected, flat-mounted eyes were captured after in situ hybridization and imported into ImageJ 1.32j (http://rsb.info.nih.gov/ij/). Using the “Analyze>Mean” option, mean intensities in eight axial, 30-µm^2^ regions were measured and further analyzed with Microsoft Excel. Analysis was done on identically processed embryos.

### Antibody Staining and Quantification of Cell Proliferation

The following primary antibodies were used: anti-caspase3 (1∶500, Abcam), anti-Phospho-Histone H3 (PH3) (1∶500, Upstate Biotechnology), anti-aPKC (1∶500, Santa Cruz Biotechnology), anti-BrdU (1∶200, Roche), and anti-ZO1 (1∶500, Zymed/Invitrogen). The secondary antibodies used are: Alexa Fluor 488 goat anti-rabbit, Alexa Fluor 488 goat anti-mouse, and Alexa Fluor 633 anti-mouse (all 1∶1,000, Invitrogen). Counterstaining was done using DAPI (1 µg/ml, Invitrogen) and Alexa Fluor 564 phalloidin (1∶400, Invitrogen). For counting PH3^+^ cells in optic vesicles, forebrains were dissected from embryos after staining and transversely split into an anterior and posterior half, which were then flat-mounted. PH3^+^ cells in stacks of consecutive, transverse, single optical sections, captured at 3-8-µm intervals, were counted according to their localization in the evaginating vesicle. Data and statistical analysis for quantification of PH3 was carried out using MS Excel and a two-tailed Student *t*-test.

### BrdU Incorporation and Detection

BrdU treatments were done by incubating the embryos in 5 mg/ml BrdU and 15% DMSO, for 20 min on ice followed by 10 min at 28.5°C just prior to fixation. BrdU detection was done as previously described [70].

### Microscopy

Live embryos were imaged with an upright Leica TCS SP5 confocal microscope using a 63× dipping lens after immobilization in 1.2% LMP agarose in embryo medium. A 40× UV-corrected lens was used for imaging fluorescently stained embryos. Image analysis and assembly was done with ImageJ, Metamorph, Volocity, and Leica LAS software. Injection of 25–50 pg in vitro–transcribed palmitoylated mRFP [71] or lynGFP RNA/embryo was used as in vivo membrane counterstain.

### Foxd1 Nomenclature

At the time of this study, the zebrafish genome contained one gene annotated as *foxd1* (GenBank accession number: NM_131271.1) and one gene annotated as *foxd1 like* (GenBank accession number: NM_212913.1). Phylogenetic sequence analysis revealed that zebrafish *foxd1 like* is orthologous to other vertebrate *foxd1* genes, whereas zebrafish *foxd1* is orthologous to *foxd2* (Figures S11 and S12). Therefore, *foxd1 like* is referred to as *foxd1* throughout this study.

## Supporting Information

Figure S1 ***fgf24*** **expression in the olfactory placode at optic vesicle and cup stages.** (A and B) Onset of expression, anterior to the forebrain (1s). (C–F) Posterior expansion along the hinge between dorsal optic vesicle leaflet and dorsal forebrain (3- and 5ss). (G–J) Lateral spreading, anterior condensation and olfactory pit preformation (see *fgf24*-nonexpressing cells, arrowheads) at 15- and 25ss. (A, C, E, G, and I) Dorsal view, anterior to the top. (B and D) Lateral view, anterior to the left. (F, H, and J) Cross-section, dorsal to the top. Dotted outlines: optic vesicle and cup boundary (E, G, and I) or neural tube boundary (F, H, and J).
(1.30 MB TIF)

Figure S2 **Target gene expression shows active Fgf signaling in the dorsal optic vesicle leaflet.** Expression of the Fgf target genes *erm*, *pea3*, *spry2*, and *spry4* at the 10ss stage in the optic vesicle of control embryos (A–D) and embryos after FgfR-inhibitor treatment (E–H). (A) *erm* is strongly expressed in the dorsal forebrain (asterisk) and the dorsal optic vesicle leaflet (arrowhead) of the control. (E) Remnant *erm* expression is only found in the dorsal forebrain in inhibitor treated embryos (asterisk). (B) *pea3* is weakly expressed in the dorsal forebrain (asterisk) but stronger in the proximal part of the dorsal optic vesicle leaflet (arrowhead) of the control. (F) Inhibitor-treated embryos show no *pea3* expression. (C) *spry2* is strongly expressed in the dorsal forebrain (asterisk) and the proximal part of the dorsal optic vesicle leaflet (arrowhead) of the control. (G) Remnant *spry2* expression is only found in the dorsal forebrain of inhibitor treated embryos (asterisk). (D) *spry4* is weakly expressed in the dorsal forebrain (asterisk) and in the proximal part of the dorsal optic vesicle leaflet (arrowhead) of the control. (H) Inhibitor-treated embryos show no *spry4* expression. All images are cross-sections, dorsal to the top; dotted lines: neural tube boundary.
(2.11 MB TIF)

Figure S3 **In vivo imaging of clnb:GFP expression between the 5- and 10ss stages.** (A) Single images from a confocal time-lapse series of cldnb:GFP expression (green) colabeled with membrane-targeted RFP (memRFP, red), captured at 20-min intervals (cross-section through one half of the forebrain, lateral to the left and dorsal to the top, bottom right: time in hours:minutes). (B) Mean number of cldnb:GFP-expressing (cldnbGFP^+^, grey) and nonexpressing (cldnbGFP^−^, white) cells in single cross-sections (captured at a 50–70-µm depth from anterior optic vesicle tip) through the optic vesicle between 5- and 10ss (error bar: standard deviation).
(3.76 MB TIF)

Figure S4 **Quantification of cldnb:GFP-expressing cell numbers in the outer optic cup layer.** Mean number of cldnb:GFP-expressing (cldnbGFP^+^, grey) and nonexpressing (cldnbGFP^−^, white) cells in single transverse sections (captured at 40–60-µm depth from anterior optic cup edge) through the outer optic cup layer between 18- and 24ss (error bar: standard deviation).
(0.07 MB TIF)

Figure S5 **Nasal restriction of cells from the outer optic cup layer.** The DsRed2 cell clone form Figure 3C is restricted to the nasal retina of the HRAS-EGFP host at 36 h. (A) lateral view, (B) optical cross-section at a ventral (B) and medial (C) level along the nasal-temporal axis (arrowheads: autofluorescent blood vessels). n, nasal; t, temporal.
(0.68 MB TIF)

Figure S6 ***foxd1*** **expression in temporal retina progenitors during optic cup formation.** (A and B) At 10ss (A) and 15ss (B), *foxd1* expression is confined to the ventral optic vesicle leaflet. (C) At 18ss, the first *foxd1*-positive cells are found at the distal part of the forming outer optic cup layer, indicating the onset of temporal retina progenitor movement into the future neural retina. The whole ventral leaflet is expressing *foxd1*. (D) At 25ss, *foxd1* expression is found in the ventral part of the outer optic cup layer, indicating continued movement. Only the distal part of the inner optic cup layer contains *foxd1*-expressing cells (white arrowhead), indicating continued movement of temporal progenitors out of this region (black arrowheads: distal/dorsal gene expression limit, dotted lines: neural tube boundary. All images are cross-sections.
(0.84 MB TIF)

Figure S7 **Live phenotype of** ***Fgf8/3/24*** **mutant embryos at 28 h.** (A) wt control embryo, (B) *fgf8^−/−^* mutant with reduced ear and lacking cerebellum (arrowheads), (C) *fgf3^−/−^* mutant with small lens (arrowhead), (D) *fgf8^−/−^*; *fgf3^+/−^* transheterozygous embryo, lacking the cerebellum and with strongly reduced ear (arrowheads), (E) *fgf8/3^−/−^* double-mutant, lacking cerebellum and ear and with small lens (arrowheads), (F) *fgf8/24^−/−^* double mutant, showing nasally tilted eye position, lack of the cerebellum, and reduction of the ear, (G) *fgf8^−/−^*; *fgf3^+/−^* transheterozygous embryo, injected with *fgf24* MO (*fgf8/24^−/−^*; *fgf3^+/−^*), showing nasally tilted eye position, lack of the cerebellum, and strong reduction of the ear (arrowheads), (H) *fgf8/3^−/−^* double mutant injected with *fgf24* MO (*fgf8/3/24^−/−^*), showing nasally tilted eye position, lack of the cerebellum and ear (arrowheads), and (I) wt embryos treated with FgfR-inh. (wt+FgfR-Inh.), showing nasally tilted eye position, lack of cerebellum, and reduced ear (arrowheads).
(2.25 MB TIF)

Figure S8 **Apical-basal cell polarity after FgfR inhibition.** (A and B) Normal localization of the apical membrane markers aPKC (A) and ZO1 (B) at 10ss, after FgfR-inh. treatment (bottom panels) compared to control embryos (top panels). (C and D) Cell delamination in the optic vesicle after FgfR-inh. treatment (D) compared to control (C) at 15ss. Sites of delamination (arrowheads in [D]) correspond to regions where apical membrane contact between dorsal and ventral leaflet (revealed by staining for aPKC, green) is lost. Images are transverse sections, counterstained with DAPI (blue) and for F-actin (red), dorsal to the top, dotted lines: neural tube boundary. d, dorsal optic vesicle leaflet; v, ventral optic vesicle leaflet.
(2.67 MB TIF)

Figure S9 **Effect of clonal dnFgfR1 overexpression on foxg1 and foxd1 in the evaginating optic vesicle.** Clonal dnFgfr1 overexpression by transplantation and heat-shock induction of rhodamine-dextran lineage-labeled *Tg* (*hsp70l:dnfgfr1-EGFP*)*pd1* cells. dnFgfr1 overexpression in the dorsal optic vesicle leaflet (arrowheads) represses *foxg1* expression (A) and leads to ectopic *foxd1* expression (B) at 12ss. Heat shocks were given at the onset of optic vesicle evagination (1-3ss). Top panels: dorsal views, bottom panels: cross-sections with dorsal to the top, left panels: bright field, right panels: fluorescent lineage label, and dotted lines: neural tube boundary (bottom panels) or optic vesicle boundary (top panels).
(1.11 MB TIF)

Figure S10 **Foxg1 morpholino knockdown.** (A) Live image of Foxg1-cherry fusion protein expression (red) in the animal pole blastoderm in a *Tg* (*h2afv:GFP*)*kca66* embryo (green) at sphere stage shows nuclear (arrowhead) and cytoplasmic localization of the protein. (B) Compared to a noninjected control (left), injection of foxg1 morpholino (foxg1MO, right) results in complete and specific depletion of the fluorescent foxg1cherry signal (red) compared to *Tg* (*h2afv:GFP*)*kca66* (h2aGFP, green) in live embryos at sphere stage (lateral views, animal to the top).
(0.64 MB TIF)

Figure S11 **Phylogenetic tree analysis of vertebrate Foxd1/2/3.** Maximum likelihood phylogeny of Foxd1/2/3 sequences from *Homo sapiens*, *Mus musculus*, *Gallus gallus*, *Xenopus laevis*, and *Danio rerio* as determined by PHYML (Guindon and Gascuel, 2003 [72]) shows that zebrafish Foxd1-like (light red shading) is orthologous to other vertebrate Foxd1 genes, whereas zebrafish Foxd1 (light blue shading) is orthologous to other vertebrate Foxd2 genes (PHYML parameters if not default: bootstrapping = 1,000 pseudo datasets; transition ratio and proportion of invariable sites = estimated; number of substitution categories = 8; gamma distribution parameter = estimated; only bootstrap values>900 are shown, based on alignment in Figure S12).
(0.14 MB TIF)

Figure S12 **Trimmed multiple sequence alignment of vertebrate Foxd1/2/3 proteins.** A multiple alignment of known zebrafish, human, mouse, chick, and frog Foxd1/2/3 protein sequences was calculated with MAFFT (Katoh et al., 2005 [73]) (623 aligned amino acids [aa]) and trimmed with GBlocks (Talavera and Castresana, 2007 [74]) (232 aligned aa) before phylogenetic tree calculation (see Figure S11). The following GenBank sequences were used: *D. rerio* Foxd1-like: NP_998078, *D. rerio* Foxd1: NP_571346, *D. rerio* Foxd3: NP_571365, *H. sapiens* Foxd3: NP_036315, *H. sapiens* Foxd1: NP_004463, *H. sapiens* Foxd2: NP_004465, *M. musculus* Foxd1: NP_032268, *M. musculus* Foxd2: NP_032619, *M. musculus* Foxd3: NP_034555, *X. laevis* Foxd1: NP_001079052, *X. laevis* Foxd2: NP_001079322, *X. laevis* Foxd3: NP_001079026, *G. gallus* Foxd2: NP_990283, *G. gallus* Foxd1: NP_990523, *G. gallus* Foxd3: NP_990282. FH, forkhead box.
(1.13 MB TIF)

Video S1 **Dynamic restriction of cldnb:GFP-expressing cells in the optic cup.** A *Tg* (*-8.0cldnb:lynGFP*)*zf106* transgenic embryo (green), colabeled with membrane-targeted RFP (red) was imaged between 18- and 24ss. Cells expressing cldnb:GFP first cover the complete outer optic cup layer and then get gradually localized to the dorsal half – the future nasal retina. Cross-section through one half of the forebrain, dorsal is up and lateral to the left. Frame interval: 10 min. Number of frames: 17. Each frame is a single confocal section.
(0.45 MB MOV)

Video S2 **Gradual displacement of single nasal retina progenitors in the optic cup.** A *Tg* (*Bactin:HRAS-EGFP*)*vu119* transgenic embryo (green), with a clone of DsRed2 (red) expressing cells in the outer optic cup layer was imaged between 18- and 21ss. Cells expressing DsRed2 first are scattered throughout the complete outer optic cup layer and then get gradually localized to the dorsal half – the future nasal retina. cross-section through one half of the forebrain, dorsal is up and lateral to the left. Frame interval: 5 min. Number of frames: 17. Each frame is a single confocal section.
(0.57 MB MOV)

## Abbreviations

RGC: retinal ganglion cell
ss: somite stage
wt: wild-type

## References

[1] Luo L, Flanagan J. G (2007). Development of continuous and discrete neural maps.. Neuron.

[2] McLaughlin T, O'Leary D. D (2005). Molecular gradients and development of retinotopic maps.. Annu Rev Neurosci.

[3] Cheng H. J, Nakamoto M, Bergemann A. D, Flanagan J. G (1995). Complementary gradients in expression and binding of ELF-1 and Mek4 in development of the topographic retinotectal projection map.. Cell.

[4] Drescher U, Kremoser C, Handwerker C, Loschinger J, Noda M (1995). In vitro guidance of retinal ganglion cell axons by RAGS, a 25 kDa tectal protein related to ligands for Eph receptor tyrosine kinases.. Cell.

[5] Holash J. A, Pasquale E. B (1995). Polarized expression of the receptor protein tyrosine kinase Cek5 in the developing avian visual system.. Dev Biol.

[6] Hindges R, McLaughlin T, Genoud N, Henkemeyer M, O'Leary D. D (2002). EphB forward signaling controls directional branch extension and arborization required for dorsal-ventral retinotopic mapping.. Neuron.

[7] Mann F, Ray S, Harris W, Holt C (2002). Topographic mapping in dorsoventral axis of the Xenopus retinotectal system depends on signaling through ephrin-B ligands.. Neuron.

[8] Schmitt A. M, Shi J, Wolf A. M, Lu C. C, King L. A (2006). Wnt-Ryk signalling mediates medial-lateral retinotectal topographic mapping.. Nature.

[9] Yuasa J, Hirano S, Yamagata M, Noda M (1996). Visual projection map specified by topographic expression of transcription factors in the retina.. Nature.

[10] Takahashi H, Shintani T, Sakuta H, Noda M (2003). CBF1 controls the retinotectal topographical map along the anteroposterior axis through multiple mechanisms.. Development.

[11] Yamagata M, Mai A, Pollerberg G. E, Noda M (1999). Regulatory interrelations among topographic molecules CBF1, CBF2 and EphA3 in the developing chick retina.. Dev Growth Differ.

[12] Schulte D, Cepko C. L (2000). Two homeobox genes define the domain of EphA3 expression in the developing chick retina.. Development.

[13] Hirose Y, Varga Z. M, Kondoh H, Furutani-Seiki M (2004). Single cell lineage and regionalization of cell populations during Medaka neurulation.. Development.

[14] England S. J, Blanchard G. B, Mahadevan L, Adams R. J (2006). A dynamic fate map of the forebrain shows how vertebrate eyes form and explains two causes of cyclopia.. Development.

[15] Wilson S. W, Houart C (2004). Early steps in the development of the forebrain.. Dev Cell.

[16] Chuang J. C, Raymond P. A (2002). Embryonic origin of the eyes in teleost fish.. Bioessays.

[17] Varga Z. M, Wegner J, Westerfield M (1999). Anterior movement of ventral diencephalic precursors separates the primordial eye field in the neural plate and requires cyclops.. Development.

[18] Rembold M, Loosli F, Adams R. J, Wittbrodt J (2006). Individual cell migration serves as the driving force for optic vesicle evagination.. Science.

[19] Li Z, Joseph N. M, Easter S. S (2000). The morphogenesis of the zebrafish eye, including a fate map of the optic vesicle.. Dev Dyn.

[20] Martinez-Morales J. R, Rembold M, Greger K, Simpson J. C, Brown K. E (2009). ojoplano-mediated basal constriction is essential for optic cup morphogenesis.. Development.

[21] Peters M. A (2002). Patterning the neural retina.. Curr Opin Neurobiol.

[22] McLaughlin T, Hindges R, O'Leary D. D (2003). Regulation of axial patterning of the retina and its topographic mapping in the brain.. Curr Opin Neurobiol.

[23] Raible F, Brand M (2004). Divide et Impera–the midbrain-hindbrain boundary and its organizer.. Trends Neurosci.

[24] Mason I (2007). Initiation to end point: the multiple roles of fibroblast growth factors in neural development.. Nat Rev Neurosci.

[25] Picker A, Brand M (2005). Fgf signals from a novel signaling center determine axial patterning of the prospective neural retina.. Development.

[26] Draper B. W, Stock D. W, Kimmel C. B (2003). Zebrafish fgf24 functions with fgf8 to promote posterior mesodermal development.. Development.

[27] Hanashima C, Shen L, Li S. C, Lai E (2002). Brain factor-1 controls the proliferation and differentiation of neocortical progenitor cells through independent mechanisms.. J Neurosci.

[28] Martynoga B, Morrison H, Price D. J, Mason J. O (2005). Foxg1 is required for specification of ventral telencephalon and region-specific regulation of dorsal telencephalic precursor proliferation and apoptosis.. Dev Biol.

[29] Xuan S, Baptista C. A, Balas G, Tao W, Soares V. C (1995). Winged helix transcription factor BF-1 is essential for the development of the cerebral hemispheres.. Neuron.

[30] Ahlgren S, Vogt P, Bronner-Fraser M (2003). Excess FoxG1 causes overgrowth of the neural tube.. J Neurobiol.

[31] Haas P, Gilmour D (2006). Chemokine signaling mediates self-organizing tissue migration in the zebrafish lateral line.. Dev Cell.

[32] Cooper M. S, Szeto D. P, Sommers-Herivel G, Topczewski J, Solnica-Krezel L (2005). Visualizing morphogenesis in transgenic zebrafish embryos using BODIPY TR methyl ester dye as a vital counterstain for GFP.. Dev Dyn.

[33] Nagayoshi S, Hayashi E, Abe G, Osato N, Asakawa K (2008). Insertional mutagenesis by the Tol2 transposon-mediated enhancer trap approach generated mutations in two developmental genes: tcf7 and synembryn-like.. Development.

[34] Lee Y, Grill S, Sanchez A, Murphy-Ryan M, Poss K. D (2005). Fgf signaling instructs position-dependent growth rate during zebrafish fin regeneration.. Development.

[35] Danesin C, Peres J. N, Johansson M, Snowden V, Cording A (2009). Integration of telencephalic Wnt and hedgehog signaling center activities by Foxg1.. Dev Cell.

[36] Dutting D, Meyer S. U (1995). Transplantations of the chick eye anlage reveal an early determination of nasotemporal polarity.. Int J Dev Biol.

[37] Schmitt E. A, Dowling J. E (1994). Early eye morphogenesis in the zebrafish, Brachydanio rerio.. J Comp Neurol.

[38] Reifers F, Bohli H, Walsh E. C, Crossley P. H, Stainier D. Y (1998). Fgf8 is mutated in zebrafish acerebellar (ace) mutants and is required for maintenance of midbrain-hindbrain boundary development and somitogenesis.. Development.

[39] Furthauer M, Reifers F, Brand M, Thisse B, Thisse C (2001). sprouty4 acts in vivo as a feedback-induced antagonist of FGF signaling in zebrafish.. Development.

[40] Whitlock K. E, Westerfield M (2000). The olfactory placodes of the zebrafish form by convergence of cellular fields at the edge of the neural plate.. Development.

[41] Scholpp S, Brand M (2004). Endocytosis controls spreading and effective signaling range of Fgf8 protein.. Curr Biol.

[42] Sai X, Ladher R. K (2008). FGF signaling regulates cytoskeletal remodeling during epithelial morphogenesis.. Curr Biol.

[43] Nakayama Y, Miyake A, Nakagawa Y, Mido T, Yoshikawa M (2008). Fgf19 is required for zebrafish lens and retina development.. Dev Biol.

[44] Martinez-Morales J. R, Del Bene F, Nica G, Hammerschmidt M, Bovolenta P (2005). Differentiation of the vertebrate retina is coordinated by an FGF signaling center.. Dev Cell.

[45] Herrera E, Marcus R, Li S, Williams S. E, Erskine L (2004). Foxd1 is required for proper formation of the optic chiasm.. Development.

[46] Ye W, Shimamura K, Rubenstein J. L, Hynes M. A, Rosenthal A (1998). FGF and Shh signals control dopaminergic and serotonergic cell fate in the anterior neural plate.. Cell.

[47] Storm E. E, Garel S, Borello U, Hebert J. M, Martinez S (2006). Dose-dependent functions of Fgf8 in regulating telencephalic patterning centers.. Development.

[48] Xu Q, Mellitzer G, Robinson V, Wilkinson D. G (1999). In vivo cell sorting in complementary segmental domains mediated by Eph receptors and ephrins.. Nature.

[49] Peters M. A, Cepko C. L (2002). The dorsal-ventral axis of the neural retina is divided into multiple domains of restricted gene expression which exhibit features of lineage compartments.. Dev Biol.

[50] Diez del Corral R, Storey K. G (2004). Opposing FGF and retinoid pathways: a signalling switch that controls differentiation and patterning onset in the extending vertebrate body axis.. Bioessays.

[51] Schock F, Perrimon N (2002). Molecular mechanisms of epithelial morphogenesis.. Annu Rev Cell Dev Biol.

[52] Gierer A (1977). Physical aspects of tissue evagination and biological form.. Q Rev Biophys.

[53] Regad T, Roth M, Bredenkamp N, Illing N, Papalopulu N (2007). The neural progenitor-specifying activity of FoxG1 is antagonistically regulated by CKI and FGF.. Nat Cell Biol.

[54] Lecaudey V, Cakan-Akdogan G, Norton W. H, Gilmour D (2008). Dynamic Fgf signaling couples morphogenesis and migration in the zebrafish lateral line primordium.. Development.

[55] Nechiporuk A, Raible D. W (2008). FGF-dependent mechanosensory organ patterning in zebrafish.. Science.

[56] Regan J. C, Concha M. L, Roussigne M, Russell C, Wilson S. W (2009). An Fgf8-dependent bistable cell migratory event establishes CNS asymmetry.. Neuron.

[57] Dormann D, Weijer C. J (2003). Chemotactic cell movement during development.. Curr Opin Genet Dev.

[58] Malicki J, Jo H, Pujic Z (2003). Zebrafish N-cadherin, encoded by the glass onion locus, plays an essential role in retinal patterning.. Dev Biol.

[59] Pujic Z, Malicki J (2001). Mutation of the zebrafish glass onion locus causes early cell-nonautonomous loss of neuroepithelial integrity followed by severe neuronal patterning defects in the retina.. Dev Biol.

[60] Pei Y. F, Rhodin J. A (1970). The prenatal development of the mouse eye.. Anat Rec.

[61] Schook P (1980). Morphogenetic movements during the early development of the chick eye. A light microscopic and spatial reconstructive study.. Acta Morphol Neerl Scand.

[62] Pombero A, Martinez S (2009). Telencephalic morphogenesis during the process of neurulation: an experimental study using quail-chick chimeras.. J Comp Neurol.

[63] Holt C (1980). Cell movements in Xenopus eye development.. Nature.

[64] Westerfield M (2000). The zebrafish book. A guide for the laboratory use of zebrafish (Danio rerio).

[65] Fischer S, Draper B. W, Neumann C. J (2003). The zebrafish fgf24 mutant identifies an additional level of Fgf signaling involved in vertebrate forelimb initiation.. Development.

[66] Herzog W, Sonntag C, von der Hardt S, Roehl H. H, Varga Z. M (2004). Fgf3 signaling from the ventral diencephalon is required for early specification and subsequent survival of the zebrafish adenohypophysis.. Development.

[67] Pauls S, Geldmacher-Voss B, Campos-Ortega J. A (2001). A zebrafish histone variant H2A.F/Z and a transgenic H2A.F/Z:GFP fusion protein for in vivo studies of embryonic development.. Dev Genes Evol.

[68] Hans S, Kaslin J, Freudenreich D, Brand M (2009). Temporally-controlled site-specific recombination in zebrafish.. PLoS One.

[69] Duggan C. D, Demaria S, Baudhuin A, Stafford D, Ngai J (2008). Foxg1 is required for development of the vertebrate olfactory system.. J Neurosci.

[70] Park H. C, Appel B (2003). Delta-Notch signaling regulates oligodendrocyte specification.. Development.

[71] Iioka H, Ueno N, Kinoshita N (2004). Essential role of MARCKS in cortical actin dynamics during gastrulation movements.. J Cell Biol.

[72] Guindon S, Gascuel O (2003). A simple, fast, and accurate algorithm to estimate large phylogenies by maximum likelihood.. Syst Biol.

[73] Katoh K, Kuma K, Miyata T, Toh H (2005). Improvement in the accuracy of multiple sequence alignment program MAFFT.. Genome Inform.

[74] Talavera G, Castresana J (2007). Improvement of phylogenies after removing divergent and ambiguously aligned blocks from protein sequence alignments.. Syst Biol.

