# Supplementary figures and images for: Dynamic Coupling of Pattern Formation and Morphogenesis in the Developing Vertebrate Retina
Source: PLoS Biol. 2009 Oct 13;7(10):e1000214. doi: 10.1371/journal.pbio.1000214 (PMC2751823; doi:10.1371/journal.pbio.1000214)

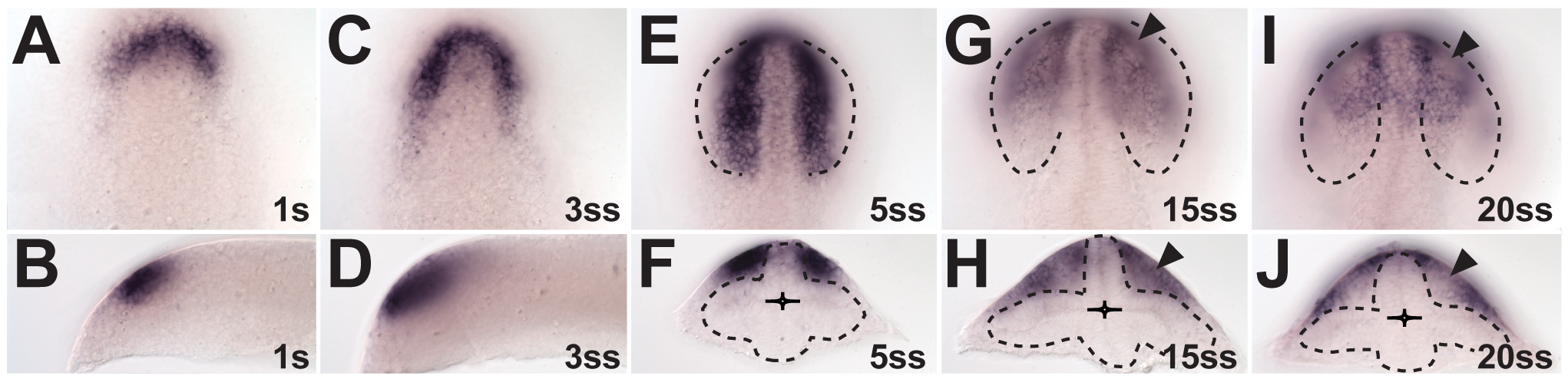

Supplement: Figure S1 — fgf24 expression in the olfactory placode at optic vesicle and cup stages. (A and B) Onset of expression, anterior to the forebrain (1s). (C–F) Posterior expansion along the hinge between dorsal optic vesicle leaflet and dorsal forebrain (3- and 5ss). (G–J) Lateral spreading, anterior condensation and olfactory pit preformation (see fgf24-nonexpressing cells, arrowheads) at 15- and 25ss. (A, C, E, G, and I) Dorsal view, anterior to the top. (B and D) Lateral view, anterior to the left. (F, H, and J) Cross-section, dorsal to the top. Dotted outlines: optic vesicle and cup boundary (E, G, and I) or neural tube boundary (F, H, and J). (1.30 MB TIF) [file pbio.1000214.s001.tif]

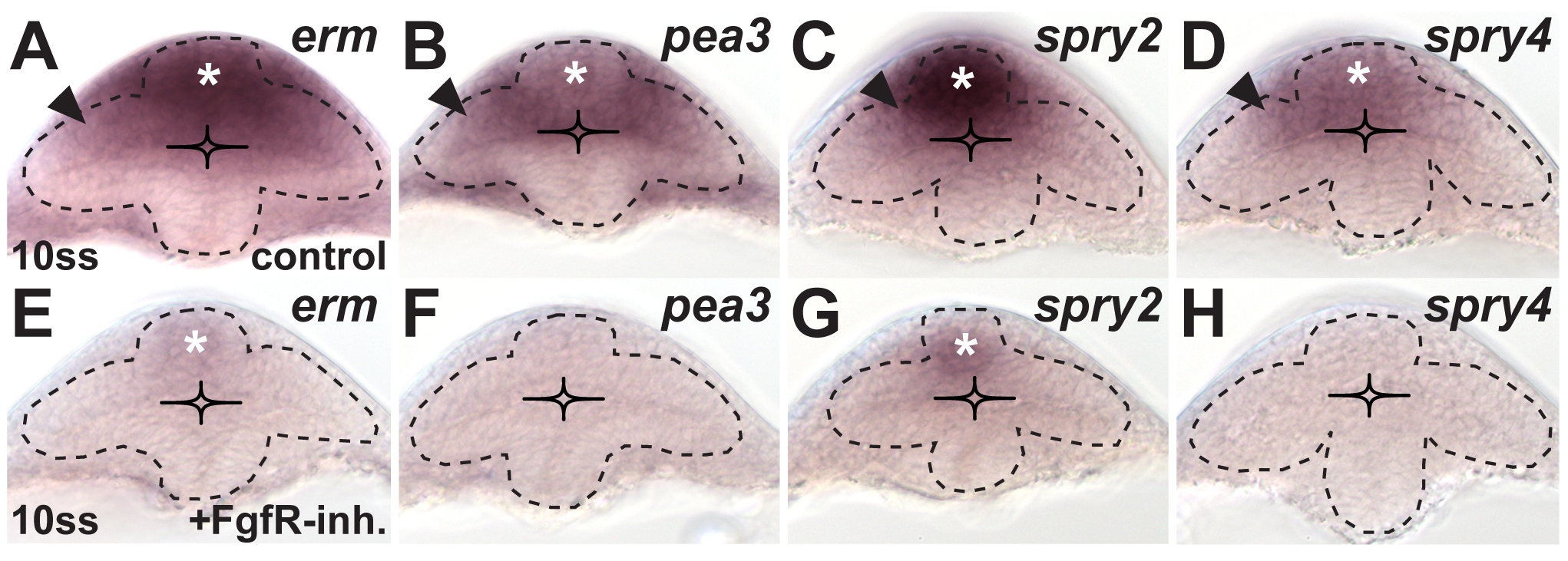

Supplement: Figure S2 — Target gene expression shows active Fgf signaling in the dorsal optic vesicle leaflet. Expression of the Fgf target genes erm, pea3, spry2, and spry4 at the 10ss stage in the optic vesicle of control embryos (A–D) and embryos after FgfR-inhibitor treatment (E–H). (A) erm is strongly expressed in the dorsal forebrain (asterisk) and the dorsal optic vesicle leaflet (arrowhead) of the control. (E) Remnant erm expression is only found in the dorsal forebrain in inhibitor treated embryos (asterisk). (B) pea3 is weakly expressed in the dorsal forebrain (asterisk) but stronger in the proximal part of the dorsal optic vesicle leaflet (arrowhead) of the control. (F) Inhibitor-treated embryos show no pea3 expression. (C) spry2 is strongly expressed in the dorsal forebrain (asterisk) and the proximal part of the dorsal optic vesicle leaflet (arrowhead) of the control. (G) Remnant spry2 expression is only found in the dorsal forebrain of inhibitor treated embryos (asterisk). (D) spry4 is weakly expressed in the dorsal forebrain (asterisk) and in the proximal part of the dorsal optic vesicle leaflet (arrowhead) of the control. (H) Inhibitor-treated embryos show no spry4 expression. All images are cross-sections, dorsal to the top; dotted lines: neural tube boundary. (2.11 MB TIF) [file pbio.1000214.s002.tif]

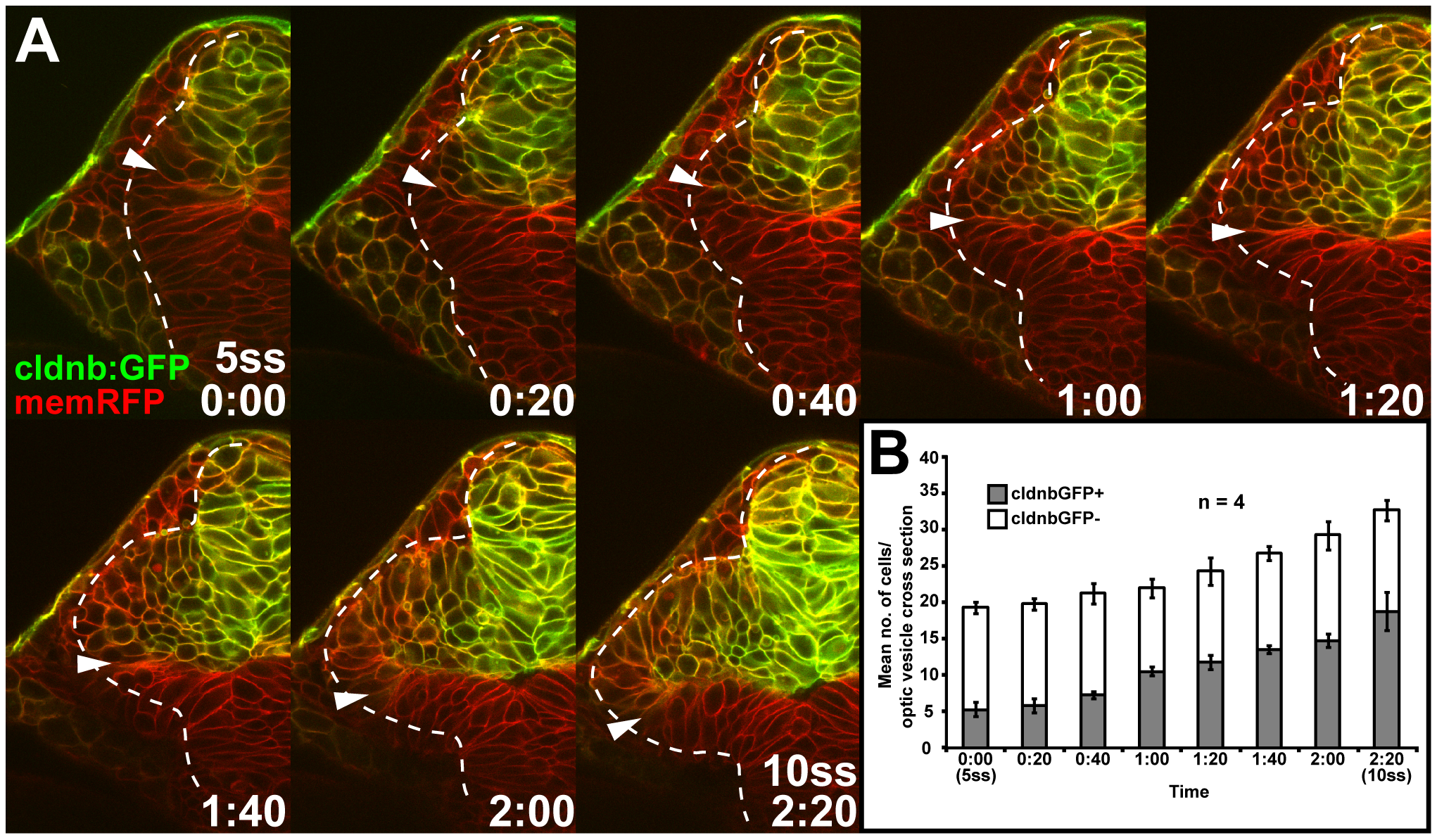

Supplement: Figure S3 — In vivo imaging of clnb:GFP expression between the 5- and 10ss stages. (A) Single images from a confocal time-lapse series of cldnb:GFP expression (green) colabeled with membrane-targeted RFP (memRFP, red), captured at 20-min intervals (cross-section through one half of the forebrain, lateral to the left and dorsal to the top, bottom right: time in hours:minutes). (B) Mean number of cldnb:GFP-expressing (cldnbGFP+, grey) and nonexpressing (cldnbGFP−, white) cells in single cross-sections (captured at a 50–70-µm depth from anterior optic vesicle tip) through the optic vesicle between 5- and 10ss (error bar: standard deviation). (3.76 MB TIF) [file pbio.1000214.s003.tif]

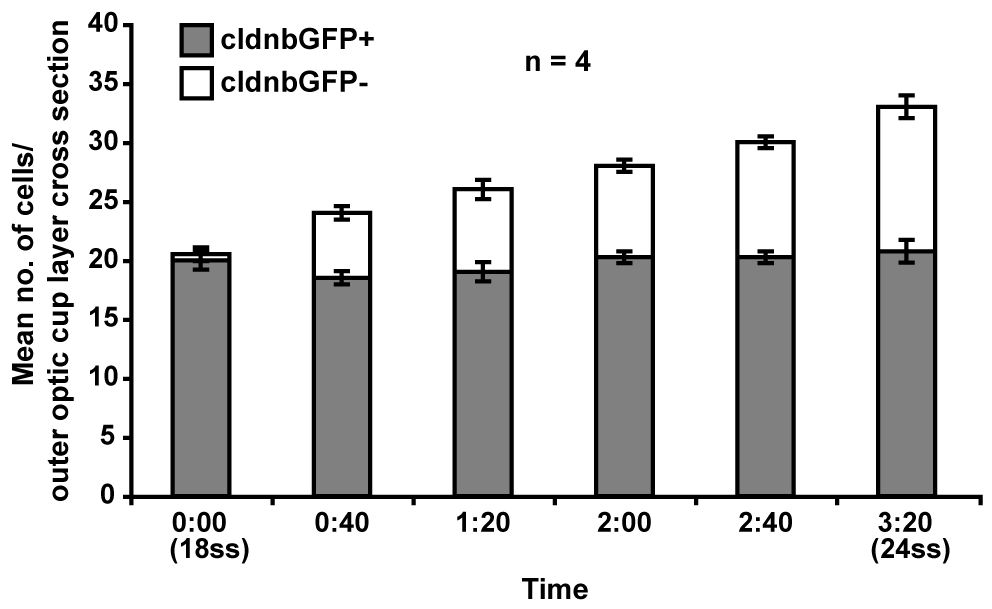

Supplement: Figure S4 — Quantification of cldnb:GFP-expressing cell numbers in the outer optic cup layer. Mean number of cldnb:GFP-expressing (cldnbGFP+, grey) and nonexpressing (cldnbGFP−, white) cells in single transverse sections (captured at 40–60-µm depth from anterior optic cup edge) through the outer optic cup layer between 18- and 24ss (error bar: standard deviation). (0.07 MB TIF) [file pbio.1000214.s004.tif]

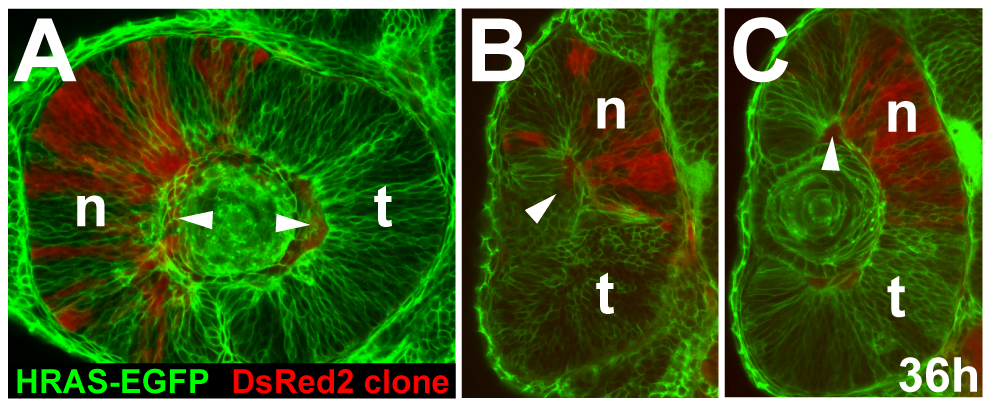

Supplement: Figure S5 — Nasal restriction of cells from the outer optic cup layer. The DsRed2 cell clone form Figure 3C is restricted to the nasal retina of the HRAS-EGFP host at 36 h. (A) lateral view, (B) optical cross-section at a ventral (B) and medial (C) level along the nasal-temporal axis (arrowheads: autofluorescent blood vessels). n, nasal; t, temporal. (0.68 MB TIF) [file pbio.1000214.s005.tif]

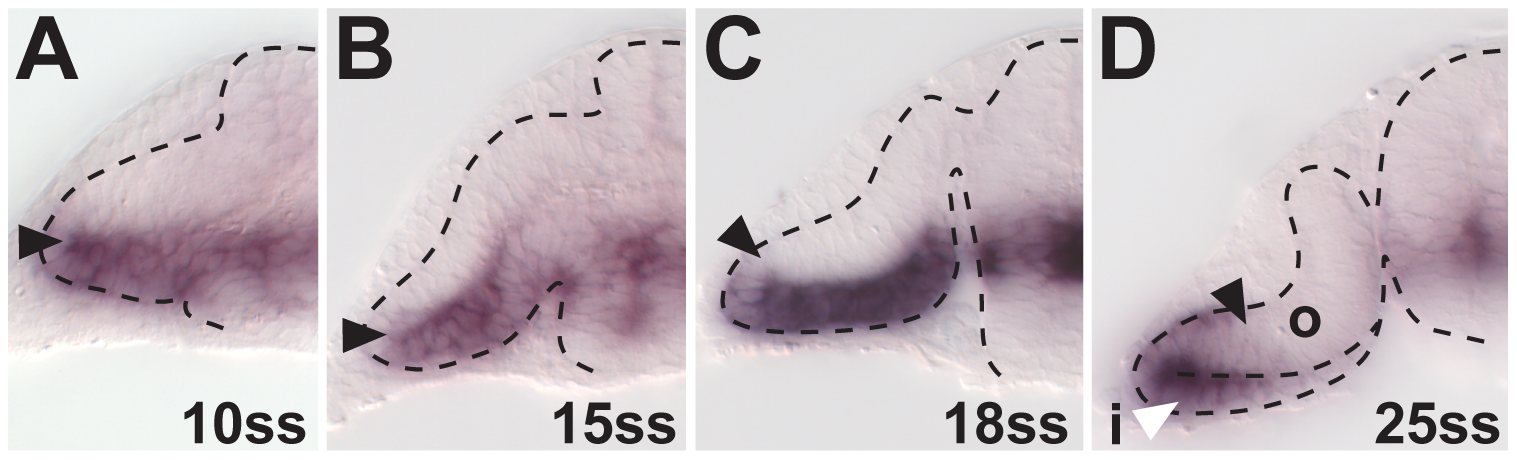

Supplement: Figure S6 — foxd1 expression in temporal retina progenitors during optic cup formation. (A and B) At 10ss (A) and 15ss (B), foxd1 expression is confined to the ventral optic vesicle leaflet. (C) At 18ss, the first foxd1-positive cells are found at the distal part of the forming outer optic cup layer, indicating the onset of temporal retina progenitor movement into the future neural retina. The whole ventral leaflet is expressing foxd1. (D) At 25ss, foxd1 expression is found in the ventral part of the outer optic cup layer, indicating continued movement. Only the distal part of the inner optic cup layer contains foxd1-expressing cells (white arrowhead), indicating continued movement of temporal progenitors out of this region (black arrowheads: distal/dorsal gene expression limit, dotted lines: neural tube boundary. All images are cross-sections. (0.84 MB TIF) [file pbio.1000214.s006.tif]

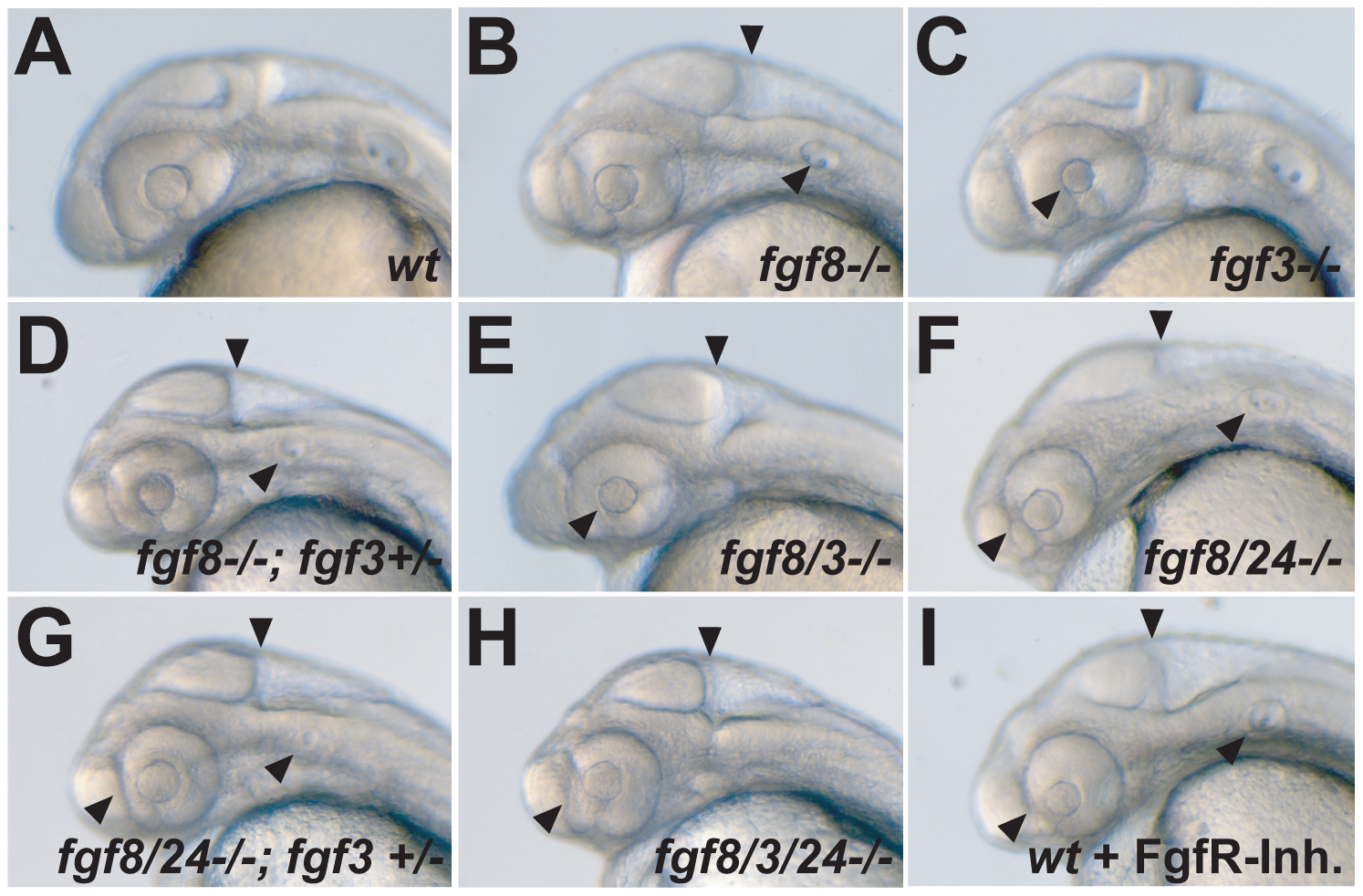

Supplement: Figure S7 — Live phenotype of Fgf8/3/24 mutant embryos at 28 h. (A) wt control embryo, (B) fgf8−/− mutant with reduced ear and lacking cerebellum (arrowheads), (C) fgf3−/− mutant with small lens (arrowhead), (D) fgf8−/−; fgf3+/− transheterozygous embryo, lacking the cerebellum and with strongly reduced ear (arrowheads), (E) fgf8/3−/− double-mutant, lacking cerebellum and ear and with small lens (arrowheads), (F) fgf8/24−/− double mutant, showing nasally tilted eye position, lack of the cerebellum, and reduction of the ear, (G) fgf8−/−; fgf3+/− transheterozygous embryo, injected with fgf24 MO (fgf8/24−/−; fgf3+/−), showing nasally tilted eye position, lack of the cerebellum, and strong reduction of the ear (arrowheads), (H) fgf8/3−/− double mutant injected with fgf24 MO (fgf8/3/24−/−), showing nasally tilted eye position, lack of the cerebellum and ear (arrowheads), and (I) wt embryos treated with FgfR-inh. (wt+FgfR-Inh.), showing nasally tilted eye position, lack of cerebellum, and reduced ear (arrowheads). (2.25 MB TIF) [file pbio.1000214.s007.tif]

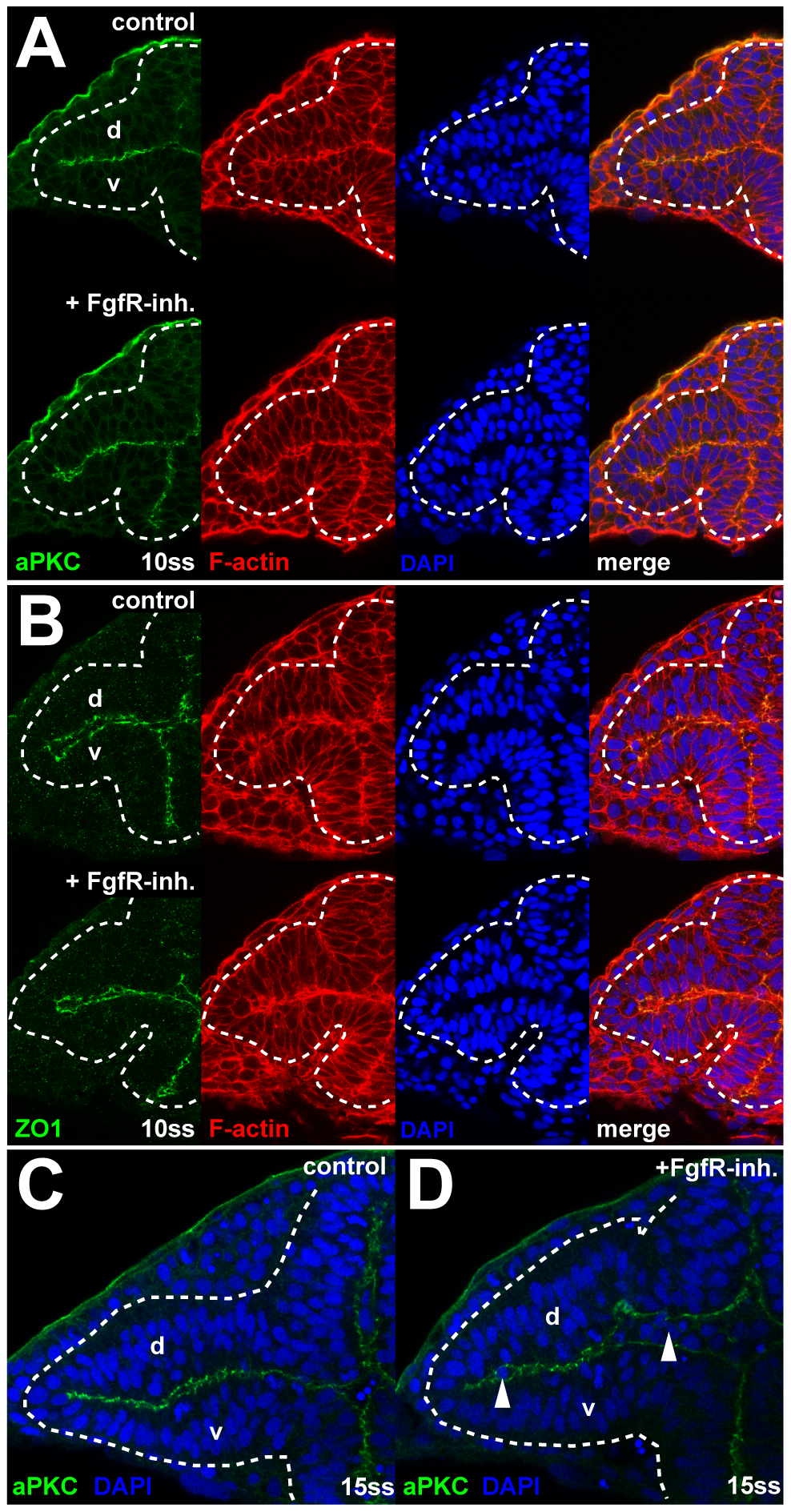

Supplement: Figure S8 — Apical-basal cell polarity after FgfR inhibition. (A and B) Normal localization of the apical membrane markers aPKC (A) and ZO1 (B) at 10ss, after FgfR-inh. treatment (bottom panels) compared to control embryos (top panels). (C and D) Cell delamination in the optic vesicle after FgfR-inh. treatment (D) compared to control (C) at 15ss. Sites of delamination (arrowheads in [D]) correspond to regions where apical membrane contact between dorsal and ventral leaflet (revealed by staining for aPKC, green) is lost. Images are transverse sections, counterstained with DAPI (blue) and for F-actin (red), dorsal to the top, dotted lines: neural tube boundary. d, dorsal optic vesicle leaflet; v, ventral optic vesicle leaflet. (2.67 MB TIF) [file pbio.1000214.s008.tif]

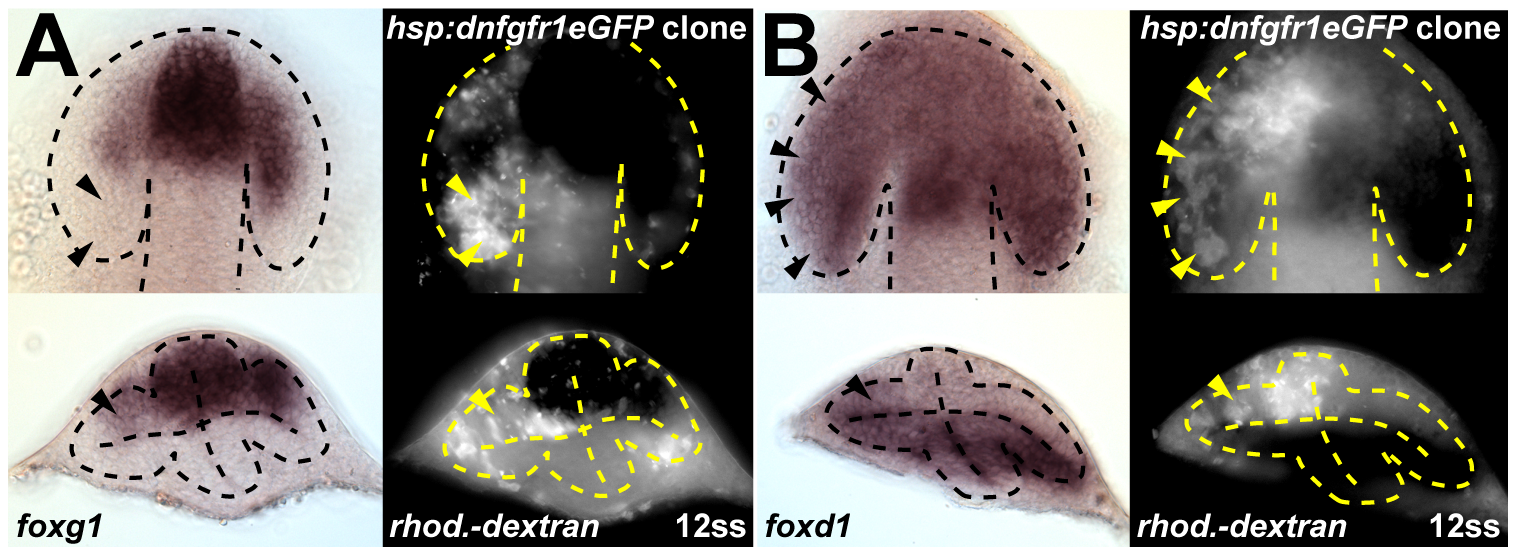

Supplement: Figure S9 — Effect of clonal dnFgfR1 overexpression on foxg1 and foxd1 in the evaginating optic vesicle. Clonal dnFgfr1 overexpression by transplantation and heat-shock induction of rhodamine-dextran lineage-labeled Tg (hsp70l:dnfgfr1-EGFP)pd1 cells. dnFgfr1 overexpression in the dorsal optic vesicle leaflet (arrowheads) represses foxg1 expression (A) and leads to ectopic foxd1 expression (B) at 12ss. Heat shocks were given at the onset of optic vesicle evagination (1-3ss). Top panels: dorsal views, bottom panels: cross-sections with dorsal to the top, left panels: bright field, right panels: fluorescent lineage label, and dotted lines: neural tube boundary (bottom panels) or optic vesicle boundary (top panels). (1.11 MB TIF) [file pbio.1000214.s009.tif]

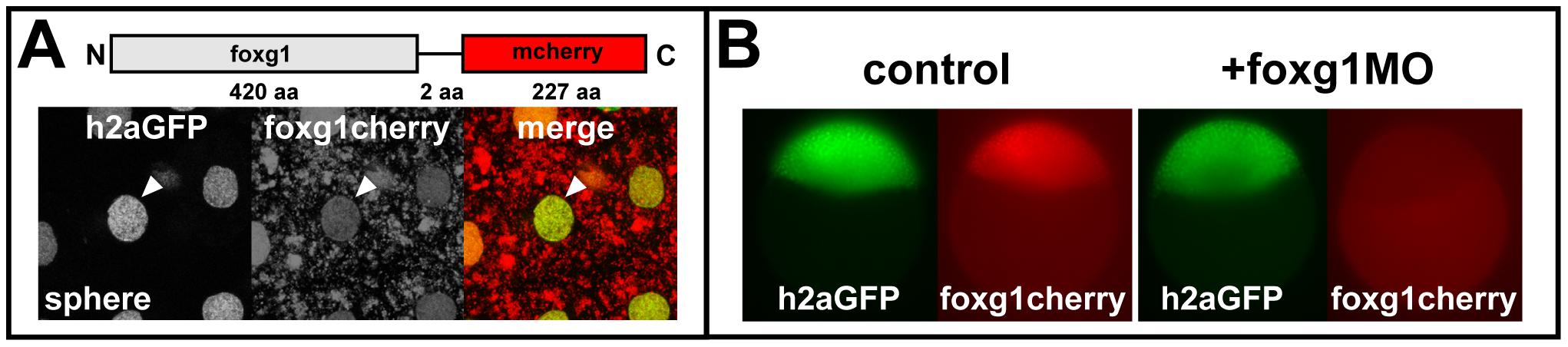

Supplement: Figure S10 — Foxg1 morpholino knockdown. (A) Live image of Foxg1-cherry fusion protein expression (red) in the animal pole blastoderm in a Tg (h2afv:GFP)kca66 embryo (green) at sphere stage shows nuclear (arrowhead) and cytoplasmic localization of the protein. (B) Compared to a noninjected control (left), injection of foxg1 morpholino (foxg1MO, right) results in complete and specific depletion of the fluorescent foxg1cherry signal (red) compared to Tg (h2afv:GFP)kca66 (h2aGFP, green) in live embryos at sphere stage (lateral views, animal to the top). (0.64 MB TIF) [file pbio.1000214.s010.tif]

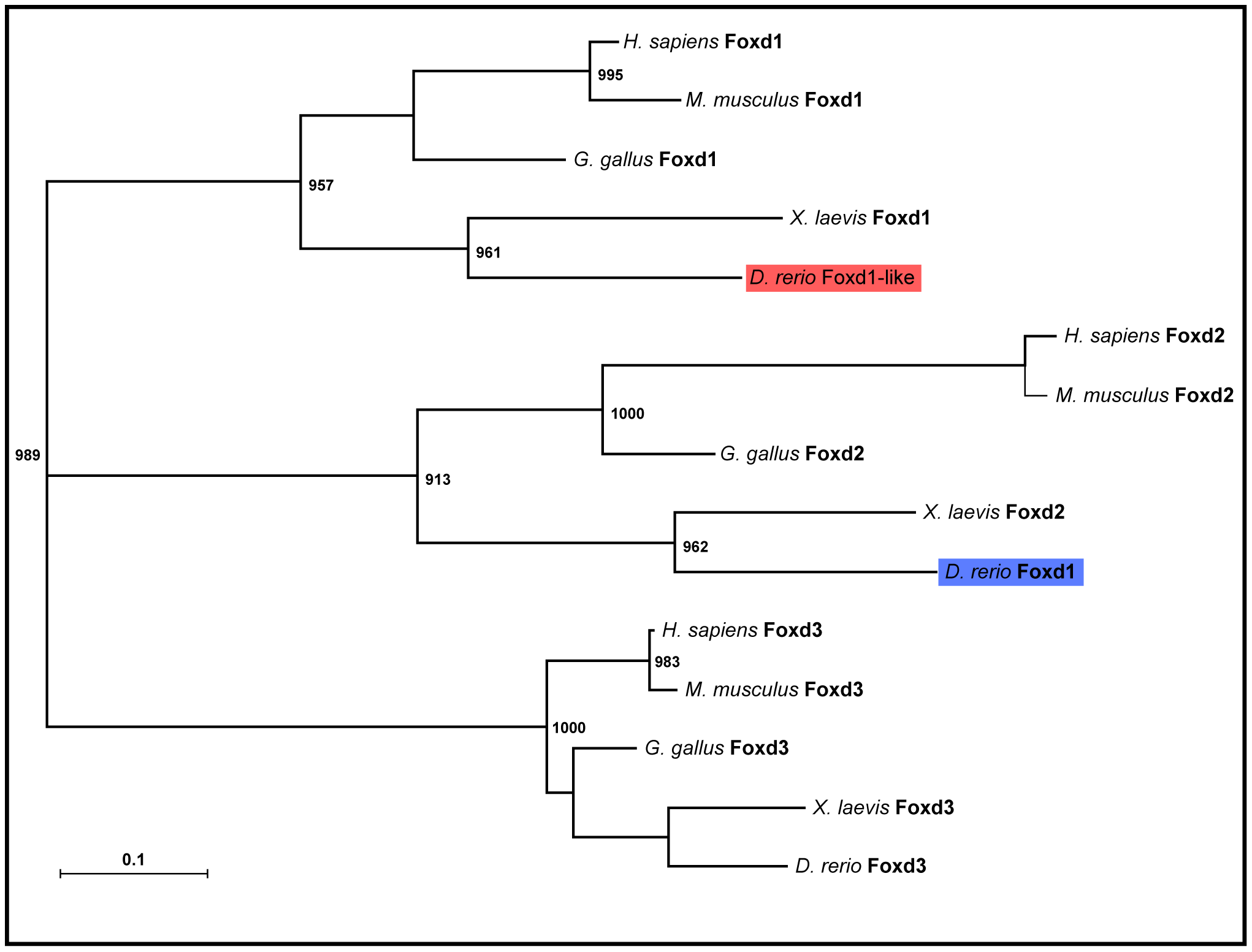

Supplement: Figure S11 — Phylogenetic tree analysis of vertebrate Foxd1/2/3. Maximum likelihood phylogeny of Foxd1/2/3 sequences from Homo sapiens, Mus musculus, Gallus gallus, Xenopus laevis, and Danio rerio as determined by PHYML (Guindon and Gascuel, 2003 [72]) shows that zebrafish Foxd1-like (light red shading) is orthologous to other vertebrate Foxd1 genes, whereas zebrafish Foxd1 (light blue shading) is orthologous to other vertebrate Foxd2 genes (PHYML parameters if not default: bootstrapping = 1,000 pseudo datasets; transition ratio and proportion of invariable sites = estimated; number of substitution categories = 8; gamma distribution parameter = estimated; only bootstrap values>900 are shown, based on alignment in Figure S12). (0.14 MB TIF) [file pbio.1000214.s011.tif]

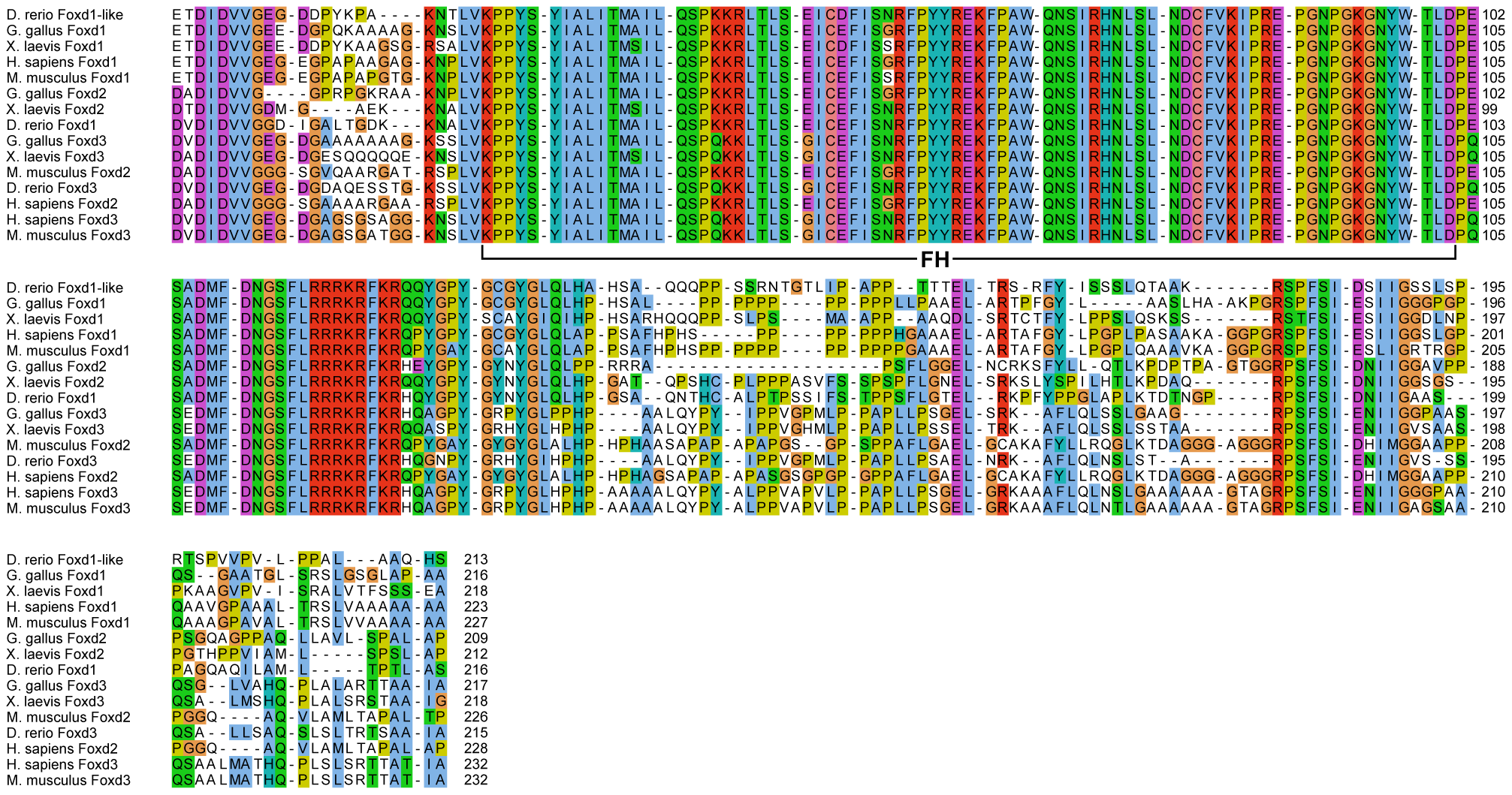

Supplement: Figure S12 — Trimmed multiple sequence alignment of vertebrate Foxd1/2/3 proteins. A multiple alignment of known zebrafish, human, mouse, chick, and frog Foxd1/2/3 protein sequences was calculated with MAFFT (Katoh et al., 2005 [73]) (623 aligned amino acids [aa]) and trimmed with GBlocks (Talavera and Castresana, 2007 [74]) (232 aligned aa) before phylogenetic tree calculation (see Figure S11). The following GenBank sequences were used: D. rerio Foxd1-like: NP_998078, D. rerio Foxd1: NP_571346, D. rerio Foxd3: NP_571365, H. sapiens Foxd3: NP_036315, H. sapiens Foxd1: NP_004463, H. sapiens Foxd2: NP_004465, M. musculus Foxd1: NP_032268, M. musculus Foxd2: NP_032619, M. musculus Foxd3: NP_034555, X. laevis Foxd1: NP_001079052, X. laevis Foxd2: NP_001079322, X. laevis Foxd3: NP_001079026, G. gallus Foxd2: NP_990283, G. gallus Foxd1: NP_990523, G. gallus Foxd3: NP_990282. FH, forkhead box. (1.13 MB TIF) [file pbio.1000214.s012.tif]
